# Molecular weight control of poly-γ-glutamic acid reveals novel insights into extracellular polymeric substance synthesis in *Bacillus licheniformis*

**DOI:** 10.1186/s13068-024-02501-9

**Published:** 2024-05-06

**Authors:** Xiaoyu Wei, Lijie Yang, Zhen Chen, Wenhao Xia, Yongbin Chen, Mingfeng Cao, Ning He

**Affiliations:** 1 Department of Chemical and Biochemical Engineering, College of Chemistry and Chemical Engineering, Xiamen University, Xiamen, 361005 Fujian China; 2 The Key Lab for Synthetic Biotechnology of Xiamen City, Xiamen University, Xiamen, People’s Republic of China; 3 College of Life Science, Xinyang Normal University, Xinyang, 464000 China

**Keywords:** *Bacillus licheniformis*, Endogenous promoter, *pgdS*, Poly-γ-glutamic acid, Molecular weight, Exopolysaccharides

## Abstract

**Background:**

The structural diversity of extracellular polymeric substances produced by microorganisms is attracting particular attention. Poly-gamma-glutamic acid (γ-PGA) is a widely studied extracellular polymeric substance from *Bacillus* species. The function of γ-PGA varies with its molecular weight (Mw).

**Results:**

Herein, different endogenous promoters in *Bacillus licheniformis* were selected to regulate the expression levels of *pgdS*, resulting in the formation of γ-PGA with Mw values ranging from 1.61 × 10^3^ to 2.03 × 10^4^ kDa. The yields of γ-PGA and exopolysaccharides (EPS) both increased in the *pgdS* engineered strain with the lowest Mw and viscosity, in which the EPS content was almost tenfold higher than that of the wild-type strain. Subsequently, the compositions of EPS from the *pgdS* engineered strain also changed. Metabolomics and RT-qPCR further revealed that improving the transportation efficiency of EPS and the regulation of carbon flow of monosaccharide synthesis could affect the EPS yield.

**Conclusions:**

Here, we present a novel insight that increased *pgdS* expression led to the degradation of γ-PGA Mw and changes in EPS composition, thereby stimulating EPS and γ-PGA production. The results indicated a close relationship between γ-PGA and EPS in *B. licheniformis* and provided an effective strategy for the controlled synthesis of extracellular polymeric substances.

**Supplementary Information:**

The online version contains supplementary material available at 10.1186/s13068-024-02501-9.

## Background

Poly-γ-glutamic acid (γ-PGA), a biopolymer composed of D- and/or L-glutamic acid units linked via gamma-amide linkages, possesses a naturally high molecular weight (Mw) [1]. Its conjugation, moisturizing, and eco-friendly properties have rendered it a promising biopolymer for various applications in the food industry, agriculture, biomedicine, and other fields [2]. Notably, the biological functions of γ-PGA can be influenced by variations in Mw and stereochemical compositions, leading to a wide range of practical application diversity [3]. For instance, low-molecular-weight (LMw)-γ-PGA has shown promise in the fields of agriculture and pharmaceuticals [4]. LMw-γ-PGA can be utilized as an antiviral biomolecule (5 kDa) [5], probiotic protectant (257 kDa) [6], and drug carrier (45–60 kDa) [7]. On the other hand, γ-PGA with a higher molecular weight (HMw) can serve as a flocculation for the removal of heavy metals (5800–6200 kDa) [8] to remove heavy metals and dyes (2500 kDa) [9], and it can serve as a scaffolding for tissue engineering (2000 kDa) [10].

γ-PGA synthetase (Pgs) is a heterotetramer consisting of PgsBCAE and is located in the cytosolic membrane [11]. The successful regulation of γ-PGA Mws has been achieved through the utilization of γ-PGA synthetases from various microbial sources. Halmschlag et al. achieved the fine-tuning of γ-PGA Mws through the integration of synthetase genes derived from various *Bacillus* strains into a single chassis microorganism (*Bacillus subtilis* PG10) [2]. The integration of *capBCAE* genes (homologues of *pgsBCAE*) from *Bacillus anthracis* enabled the production of LMw-γ-PGA with a range of 29 to 34 kDa. Additionally, the integration of *pgsBCAE* genes from *Bacillus amyloliquefaciens* resulted in the synthesis of medium-molecular-weight (MMw)-γ-PGA with a range of 170 to 660 kDa. In contrast, the natural *pgsBCAE* genes from *B. subtilis* produced γ-PGA with a much higher molecular weight, reaching up to 8500 kDa. Furthermore, three types of γ-PGA hydrolases (PgdS [12], CwlO [13], and Ggt [14]) have also been widely studied to control the Mws of γ-PGA. The mutant strains with deletion of the degradation genes *pgdS*, *ggt*, or *cwlO* all exhibited higher γ-PGA Mws compared to the wild-type *B. amyloliquefaciens* LL3 [15]. Sha et al. introduced the exogenous *pgdS* (encoding γ-PGA hydrolase) from *B. subtilis* NX-2 into *B. amyloliquefaciens* NB, resulting in the production of γ-PGA with a Mw of 20–30 kDa [16]. In the study conducted by Chen et al. [17], the native promoter of *pgdS* in *B. subtilis* KH2 was substituted with the exponential growth phase response promoter PabrB, resulting in a decrease in the Mw of γ-PGA to 411 kDa. Similarly, in *B. licheniformis* WX-02, overexpression of the *pgdS* gene led to a reduction in the Mw of γ-PGA from 1000–1200 kDa to 600–800 kDa [18]. Additionally, researchers successfully achieved the production of γ-PGA with a specific Mw of 78 kDa by optimizing signal peptides to regulate *pgdS* expression [19]. To enable dynamic regulation of Mw, Sha et al. employed CRISPRi technology to construct a regulatory system. Through the manipulation of various inducers, such as xylose, maltose, and arabinose, the expression levels of *pgdS* were regulated, resulting in hierarchical regulation of Mw ranging from 50 to 1400 kDa in *B. amyloliquefaciens* [20]. Therefore, it is feasible to produce γ-PGA with a specific Mw by controlling the expression level of *pgdS*.

Most *Bacillus* species can simultaneously synthesize at least two kinds of extracellular polymeric substances, such as *B. licheniformis* for exopolysaccharides (EPS) and γ-PGA [21] and *B. amyloliquefaciens* for levan and γ-PGA [15]. Previous research has revealed a competitive relationship between different extracellular polymeric substances in bacteria. For instance, it was reported that levan was secreted with a maximum levan titre of 22.6 g/L in the *pgsBCA* cluster knockout strain of *B. amyloliquefaciens* NK-1 [22]. Additionally, the knockout of *epsA-O* (gene clusters responsible for fructan biopolymer EPS synthesis) led to an increase in γ-PGA content from 78.6% to 95.2% in *B. amyloliquefaciens* [23]. Feng et al. discovered that the simultaneous deletion of *cwlO* and the *epsA-O* cluster led to a 63.2% increase in γ-PGA yield in *B. amyloliquefaciens* LL3 [24]. Qiu et al. employed a modular pathway strategy to enhance γ-PGA production in *B. amyloliquefaciens*. This involved overexpressing *cscA* (encoding inulin natural hydrolase), *sac* (encoding levanase), and *osC* (encoding endoinulinas), as well as key genes associated with EPS metabolism reduction (*scrk*, *pgi*, *pfkA*, *gapA*, *gapB*, and *pyk*), while also knocking out the EPS operon *epsA-O* and *cwlO* (encoding cell wall DL-endopeptidase). Consequently, the genetically modified strain exhibited a significant enhancement in γ-PGA production, reaching a level of 32.14 g/L [25]. Furthermore, it was reported that the deletion of *pgdS* in *B. subtilis* led to an increase in γ-PGA Mw from 2.23 × 10^3^ to 2.49 × 10^3^ kDa, although there was no significant increase in yield. However, the concurrent deletion of *pgdS* and *ggt* led to a substantial enhancement in γ-PGA yield, reaching 40 g/L, along with an improvement in midsize polymeric chains (0.1–2 MDa) [26]. In contrast, the increased expression of *pgdS* also led to a 54% augmentation in γ-PGA yield in *B. licheniformis* WX-02, which can be attributed to the enhanced transcription levels of glutamate transporter and γ-PGA synthetase genes [18]. Therefore, it is postulated that manipulating the expression of *pgdS* in various strains may augment the production of extracellular polymeric substances. Nevertheless, the precise mechanism underlying the synthesis of these substances and the influence of Mw on their production remain ambiguous.

Our previous research identified the presence of heteropolysaccharide, consisting of neutral sugars, amino sugars, and uronic acid, in the culture broth of *B. licheniformis* CGMCC 2876. Additionally, we observed that altering the Mw of γ-PGA influenced the production ratio of γ-PGA and EPS. In this study, we conducted a screening of various strengths of endogenous promoters in *B. licheniformis* CGMCC 2876 to control the Mw of γ-PGA. We tried to obtain γ-PGA with diverse Mws by regulating the expression of *pgdS*. Metabolomics and RT-qPCR were performed to investigate the synthesis pathways of EPS and γ-PGA in *B. licheniformis*, and an effective EPS and γ-PGA with a low Mw production system was expected to be established.

## Results

### Screening for endogenous promoters with strong expression in *B. licheniformis*

**Fig 1:**
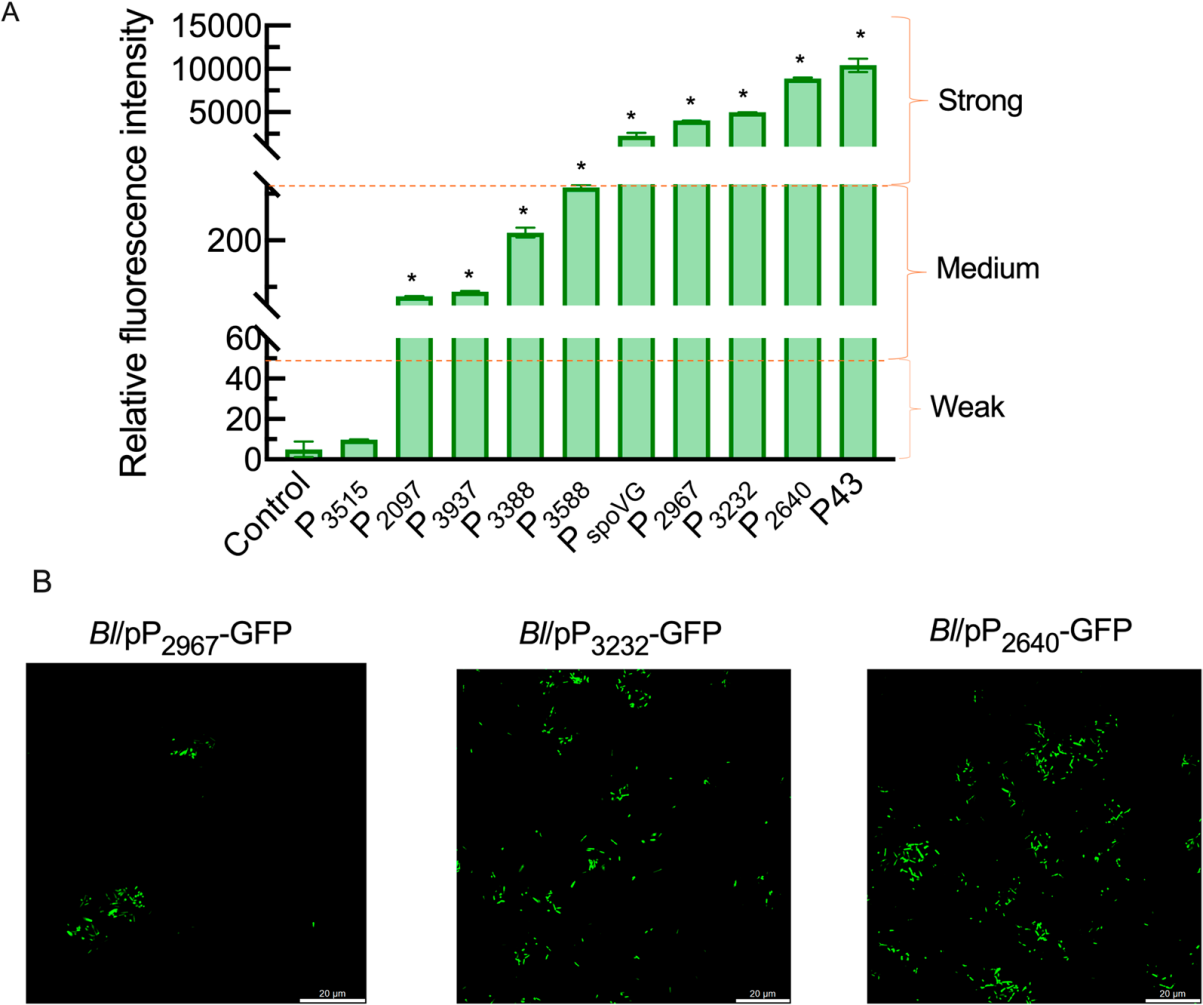
Fluorescent intensity in *B. licheniformis* with different endogenous promoter. **A** The relative fluorescence intensity of recombinant expression vectors construction for promoters in *B. licheniformis* cultured for 24 h. **B** The fluorescence scanning of recombinant cells

Promoter modification has been confirmed as an effective strategy for constructing a microbial host [27, 28]. The transcriptome of *B. licheniformis* CGMCC 2876 was used to screen the genes, which exhibited high transcription levels and located at the region 2 kb before the transcription start site. We screened 8 strong endogenous promoters, P_3937_, P_2640_, P_3588_, P_2967_, P_3232_, P_2097_, P_3388_, and P_3515_. The promoter sequence was obtained by the promoter prediction website (Additional file 1: Table S4). As shown in Fig. 1A, the highest relative fluorescence intensity of endogenous promoters reached 8.89 × 10^3^ in *Bl*/pP_2640_-GFP, and the second highest relative fluorescence intensity reached 4.98 × 10^3^ in *Bl*/pP_3232_-GFP and 4.02 × 10^3^ in *Bl*/pP_2967_-GFP. Moreover, strong fluorescence intensities were 10.46-fold higher than those of P_3388_ (2.17 × 10^2^), even over the fluorescence intensities (2.27 × 10^3^) of P_spoVG,_ which was the strong promoter in *B. subtilis* as a control [29]. The results also demonstrated that there was no correlation between the transcription strengths and GFP fluorescence intensities among these promoters, which was consistent with previous reports [27, 30]. Under the action of the endogenous promoters, strong fluorescence of *Bl*/pP_2640_-*pgdS*, *Bl*/pP_2967_-*pgdS*, and *Bl*/pP_3232_-*pgdS* was observed in bacterial morphology (Fig. 1B).

### Effects of *pgdS* gene expression levels on the Mw and yield of γ-PGA

**Fig 2:**
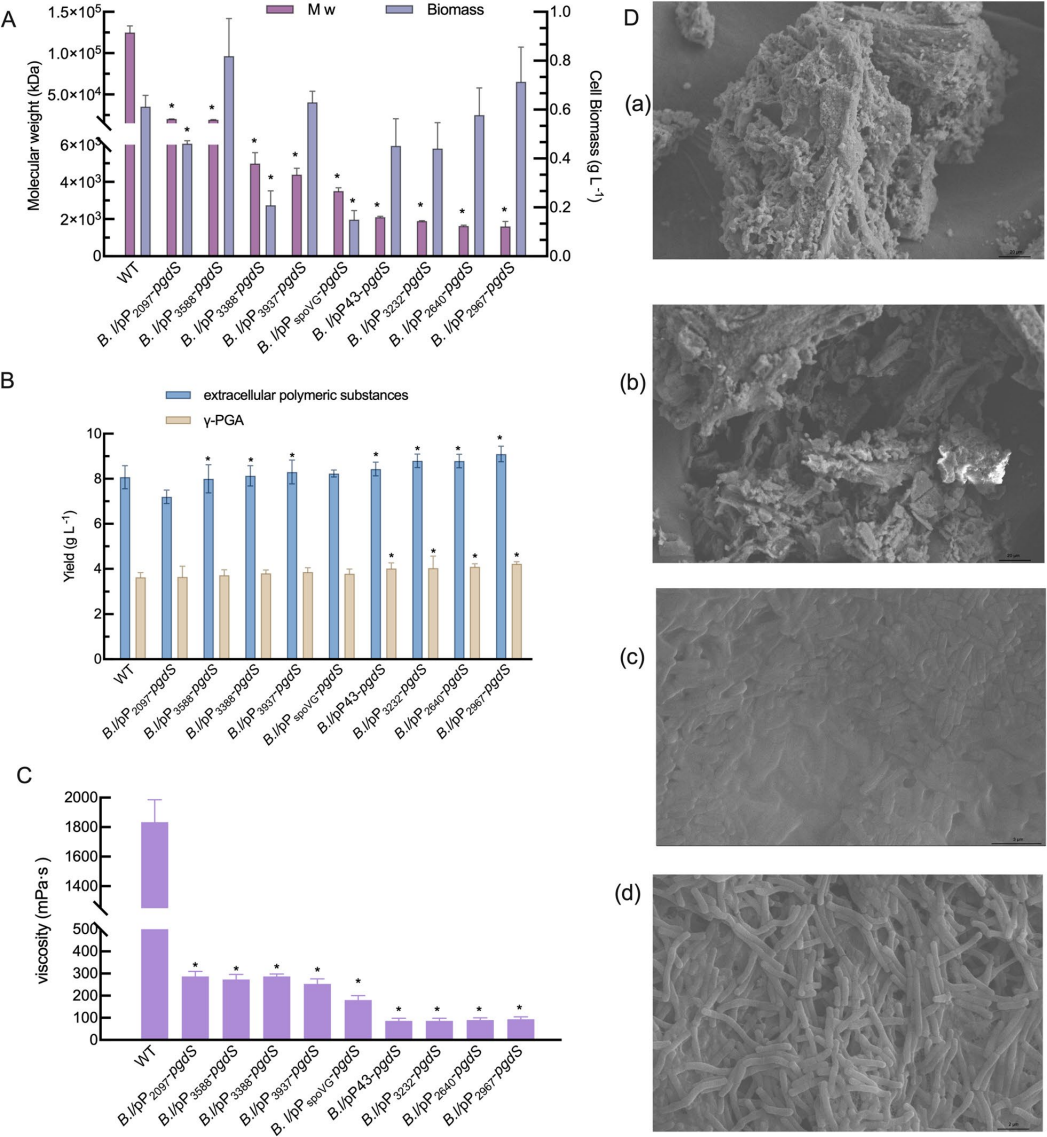
Characterization of the molecular weight of γ-PGA and cell growth in *B. licheniformis* under different promoters. **A** Mws of γ-PGA and biomass. **B** The yields of extracellular polymeric substances and γ-PGA. **C** The viscosity of extracellular polymeric substances. **D** The scanning electron microscopy of extracellular polymeric substances (a,b) and strain (c,d). The data are presented as the mean ± SD of three replications. The asterisk indicates significant differences among strains at 0.05 level (*p* < 0.05)

As indicated in Fig. 2A, enhanced overexpression of *pgdS* enabled the efficient reduction of γ-PGA Mws ranging from 2.03 × 10^4^ to 1.61 × 10^3^ kDa. The average Mw of γ-PGA from the wild-type strain was 1.25 × 10^5^ kDa. Then, three strongest promoters as tested with GFP give the highest reduction in Mws of γ-PGA. The Mws of γ-PGA produced by strain *Bl*/pP_2640_-*pgdS* and *Bl*/pP_3232_-*pgdS* were 1.63 × 10^3^ kDa and 1.89 × 10^3^ kDa. Furthermore, the Mw of γ-PGA produced by strain *Bl*/pP_2967_-*pgdS* was reduced to 1.61 × 10^3^ kDa, which were the largest decline in Mws. We found that the Mw of γ-PGA with enhanced promoter strength decreased by over 98% compared to that of the wild-type strain. The biomasses of these *pgdS* engineered strains of P_2640_, P_3232,_ and P_2967_ were approximately the same as that of the wild-type strain.

These endogenous promoters were applied to drive the expression of *pgdS* using RT-qPCR (Additional file 1: Fig. S1). The results showed that the expression levels of *pgdS* increased gradually in the recombinants compared with the wild-type strain. *Bl*/pP_2967_-*pgdS* exhibited the highest *pgdS* expression level, showing the most effective drive to start *pgdS* transcription, resulting in the lowest Mw of γ-PGA (1.61 × 10^3^ kDa). The endogenous promoters (P_3232_ and P_2640_) enhanced *pgdS* expression levels 7.59- and 7.68-fold for LMw γ-PGA (1.89 × 10^3^ and 1.63 × 10^3^ kDa). The *Bl*/pP_2097_-*pgdS* and *Bl*/pP_3588_-*pgdS* strains exhibited medium Mw γ-PGA (2.03 × 10^4^ kDa and 1.93 × 10^4^ kDa) with 1.44- and 1.93-fold expression levels of *pgdS*, respectively. Our results are consistent with previous studies showing an apparent reduction in γ-PGA Mws along with an enhanced *pgdS* expression level.

The yields of extracellular polymeric substances from the *pgdS* engineered strains were also evaluated (Fig. 2B). The yields of extracellular polymeric substances from the *Bl*/pP_3232_-*pgdS* and *Bl*/pP_2640_-*pgdS* strains were comparable (8.80 g/L and 8.78 g/L, respectively), approximately 9% higher than that from the wild-type strain. The *Bl*/pP_2640_-*pgdS* strain showed increased γ-PGA production of 4.09 g/L compared with the 3.62 g/L γ-PGA yield from the wild-type strain. *Bl*/pP_2967_-*pgdS* secreted 9.10 g/L extracellular polymeric substances with a content of 4.22 g/L γ-PGA, an increase of 12.8% and 16.54% compared with those from the wild-type strain, respectively. The results showed that along with the enhanced *pgdS* expression level (over sevenfold) by the enhanced promoter strength (P_2640_, P_2967_, P_3232_), the drastic 98% reduction in γ-PGA Mws was from 1.25 × 10^5^ kDa to 1.61 × 10^3^ kDa. In addition, we found that the yields of extracellular polymeric substances in *pgdS* recombinant strains were increased when the Mws of γ-PGA decreased by over 90% compared to the wild-type strain.

The viscosity of the extracellular polymeric substances produced by the *pgdS* engineered strains was greatly reduced (87 mPa.s to 286 mPa.s), and *B. licheniformis* CGMCC 2876 showed the highest viscosity of 1833 mPa.s (Fig. 2C). The studies conducted by Tian et al. and Dong et al. also showed increasing γ-PGA yield along with low viscosity [18, 19]. Scanning electron microscopy revealed that the extracellular polymeric substances produced by *pgdS* engineered strains were looser with a larger void between the bacteria, and the viscosity was lower. While the wild-type strain was tighter and the viscosity was larger (Fig. 2D). Collectively, a large reduction in the Mw of γ-PGA is conducive to increase the yield of extracellular polymeric substances.

### Effect of expression level of *pgdS* gene on the yield and composition of exopolysaccharides

**Fig 3:**
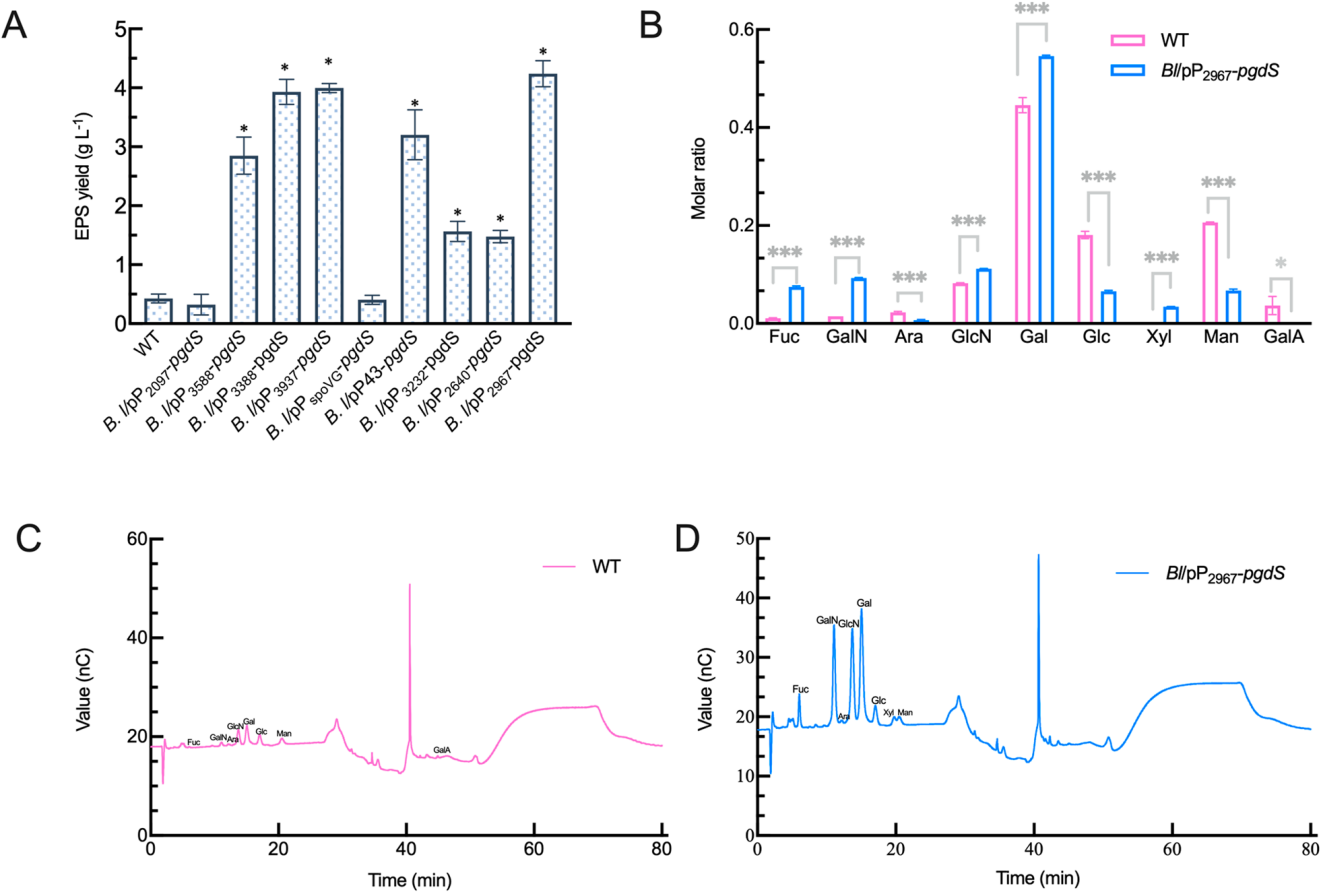
Characterization of EPS in wild-type and recombinant strains. **A** The yields of EPS in wild-type and recombinant strains. **B** The monosaccharide composition of EPS produced by wild-type and *Bl*/pP_2967_*-pgdS* strains. **C** The ion chromatograms of monosaccharides of EPS produced by wild-type strain. **D** The ion chromatograms of monosaccharides of EPS produced by *Bl*/pP_2967_*-pgdS* strain. The data are presented as the mean ± SD of three replications. The asterisk indicates significant differences among strains at 0.01 level (*p* < 0.01)

In view of the increasing γ-PGA yield along with the reduction in the Mw of γ-PGA, the yield of EPS synthesis was further studied. These recombinant strains showed varying degrees of increased EPS production (Fig. 3A). EPS yield from *Bl*/pP_3232_-*pgdS* (1.56 g/L) and *Bl*/pP_2640_- *pgdS* (1.47 g/L) was approximately 3.5-fold higher than that from the wild-type strain (0.43 g/L), while EPS yield from *Bl*/pP_2097_-*pgdS* was comparable with that of the wild-type strain. *Bl*/pP_2967_-*pgdS* led to an almost tenfold increase (4.24 g/L) in EPS production, accompanied by the largest decline in γ-PGA Mw to 1.61 × 10^3^ kDa.

We further determined the components of EPS from the *Bl*/pP_2967_-*pgdS* and wild-type strains (Fig. 3B). Specifically, the galactose content increased from 44.57 mol% to 54.60 mol%, glucosamine hydrochloride (from 8.23 mol% to 11.17 mol%), galactosamine hydrochloride (from 1.5 mol% to 9.27 mol%), and xylose (3.43 mol%) were also detected in *Bl*/pP_2967_-*pgdS,* while the proportions of mannose, glucose, and arabinose decreased. Compared with the wild-type strain in Fig. 3C, the content of fucose (1.1 mol% to 7.36 mol%) in *Bl*/pP_2967_-*pgd* increased significantly (Fig. 3D). The results showed 10.71 mol% more amino sugars (glucosamine hydrochloride and galactosamine hydrochloride), while the contents of neutral sugars decreased by 10.6 mol%. In addition, galacturonic acid (belonging to uronic acid) decreased from 3.67 mol% to 0 mol% in *Bl*/pP_2967_-*pgdS*. The results indicated that the wild-type strain and *Bl*/pP_2967_-*pgdS* were heteropolysaccharides with galactose as its main component of EPS; in particular, fucose, xylose, and glucosamine hydrochloride content in *Bl*/pP_2967_-*pgdS* was significantly increased.

### Metabolite profile analysis

**Fig 4:**
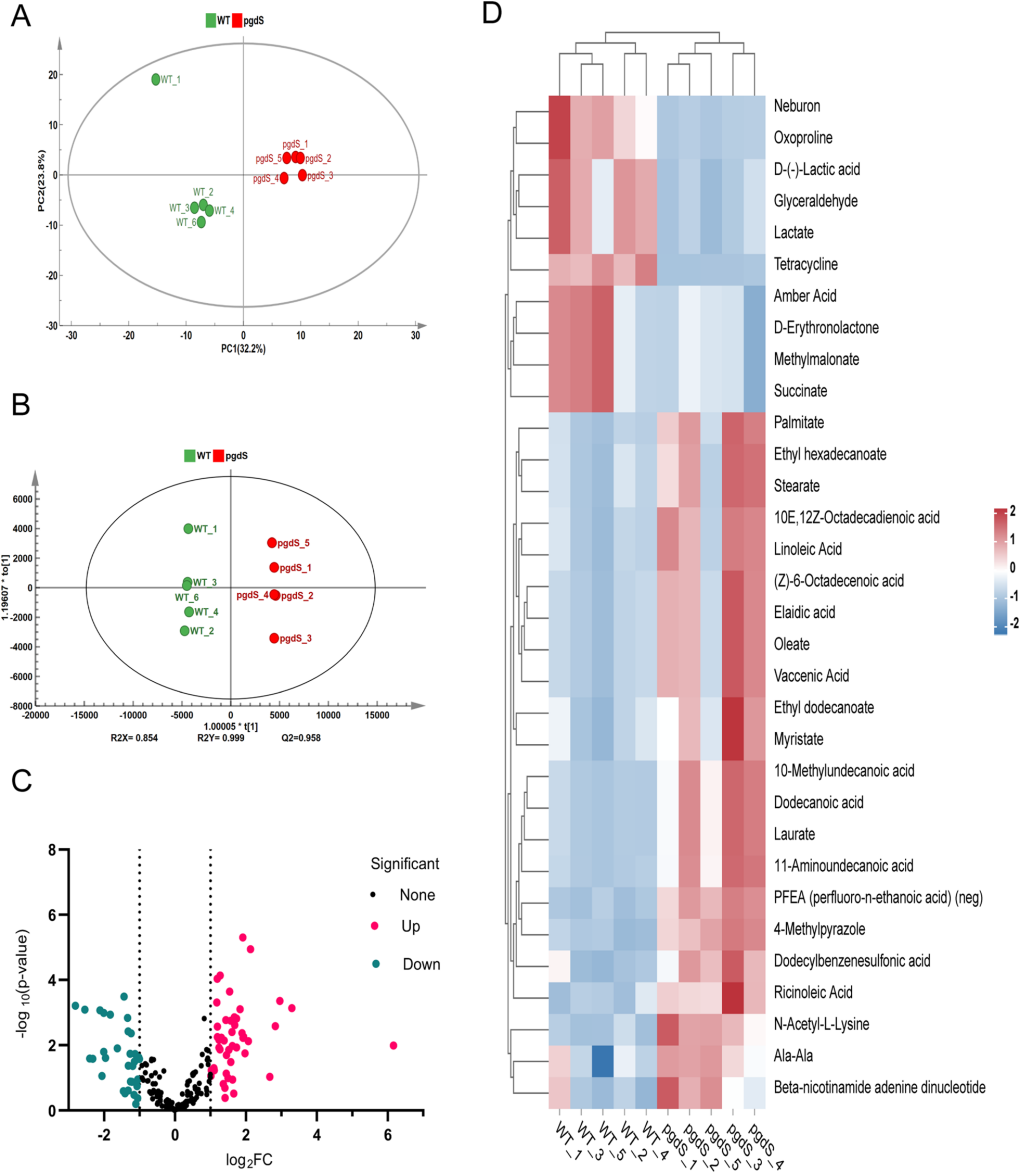
Screening and identification of differential metabolites of wild-type and *Bl*/pP_2967_*-pgdS* strains. **A** The cluster analysis of metabolites with PCA score plot. **B** The cluster analysis of with OPLS-DA. **C** The volcano plot of metabolites in *Bl/*pP_2967_*-pgdS*. **D** The heatmap of normalized concentrations of differential metabolites based on OPLS-DA results. Each column represents an individual metabolite. The normalized abundance values are depicted from blue to red, where red and blue indicate an increase and decrease, respectively. All data are expressed as the means of five replicates

The principal component analysis (PCA) score plot and orthogonal projections to latent structures (OPLS-DA) models were used for the cluster analysis of global systems of intracellular metabolites (Fig. 4A, B). The results confirmed the changes in the intracellular metabolome caused by the differences in the expression of *pgdS*. As Fig. 4A shows, the wild-type strain and *Bl/pP*_2967_*-pgdS* strain had obvious clustering. The OPLS-DA model had a better quality of screening simulations for differences between the groups and could more realistically screen the metabolite differences between the groups (Fig. 4B). The cross-validation (200 permutations) tests indicated that the OPLS-DA models contained high discrimination and predictive capability (Additional file 1: Fig. S3).

Differentially abundant metabolites were determined with variable importance in the projection (VIP) values greater than 1, and *p* values less than 0.05 were defined as statistically significant [31]. According to OPLS-DA, the VIP plot showed 78 metabolites (VIP > 1). The differentially abundant metabolites mainly included amino acids, sugar alcohols, fatty acids, and antibiotics. The *pgdS* engineered strain had 37 upregulated metabolites (fold change > 2 and *p* < 0.05) and 19 downregulated metabolites (fold change < 0.5 and *p* < 0.05) (Fig. 4C). Differentially abundant metabolites in the two strains were visually displayed in a heatmap plot (Fig. 4D). The upregulated metabolites were aspartic acid, arginine, tyrosine, proline, citrulline, and organic acids, including linoleic acid, lauric acid, and isooleic acid, as well as some antibiotic substances. The downregulated differentially abundant metabolites of aldose and sugar alcohol were glyceraldehyde, erythrose, mannitol, glucosamine, and galactosamine. In addition, correlations between these metabolites of the two strains were quantified using the Pearson correlation coefficient [32]. The correlations were visualized in colour-coded correlation matrices (Additional file 1: Fig. S2).

The differentially abundant metabolites were subjected to enrichment analysis of KEGG metabolic pathways (Additional file 1: Fig. S4). These differentially abundant metabolites mainly involved 6 KEGG metabolic pathways in negative ion mode, including biosynthesis of unsaturated fatty acids (*p* = 0.0000135), fructose and mannose metabolism (*p* = 0.0153), pyruvate metabolism (*p* = 0.0184), phenylalanine, tyrosine, and tryptophan biosynthesis (*p* = 0.0385), riboflavin metabolism (*p* = 0.0385), and linoleic acid metabolism (*p* = 0.0479). There were 7 KEGG metabolic pathways in positive ion mode, including biosynthesis of aminoacyl-tRNA (*p* = 0.0000391), phenylalanine metabolism (*p* = 0.00333), arginine and proline metabolism (*p* = 0.00422), arginine biosynthesis (*p* = 0.0066), pantothenate and CoA biosynthesis (*p* = 0.0121), glutathione metabolism (*p* = 0.0255), and riboflavin metabolism (*p* = 0.036).

### Transcriptional and metabolomic regulation of the *pgdS* engineered strain

**Fig 5:**
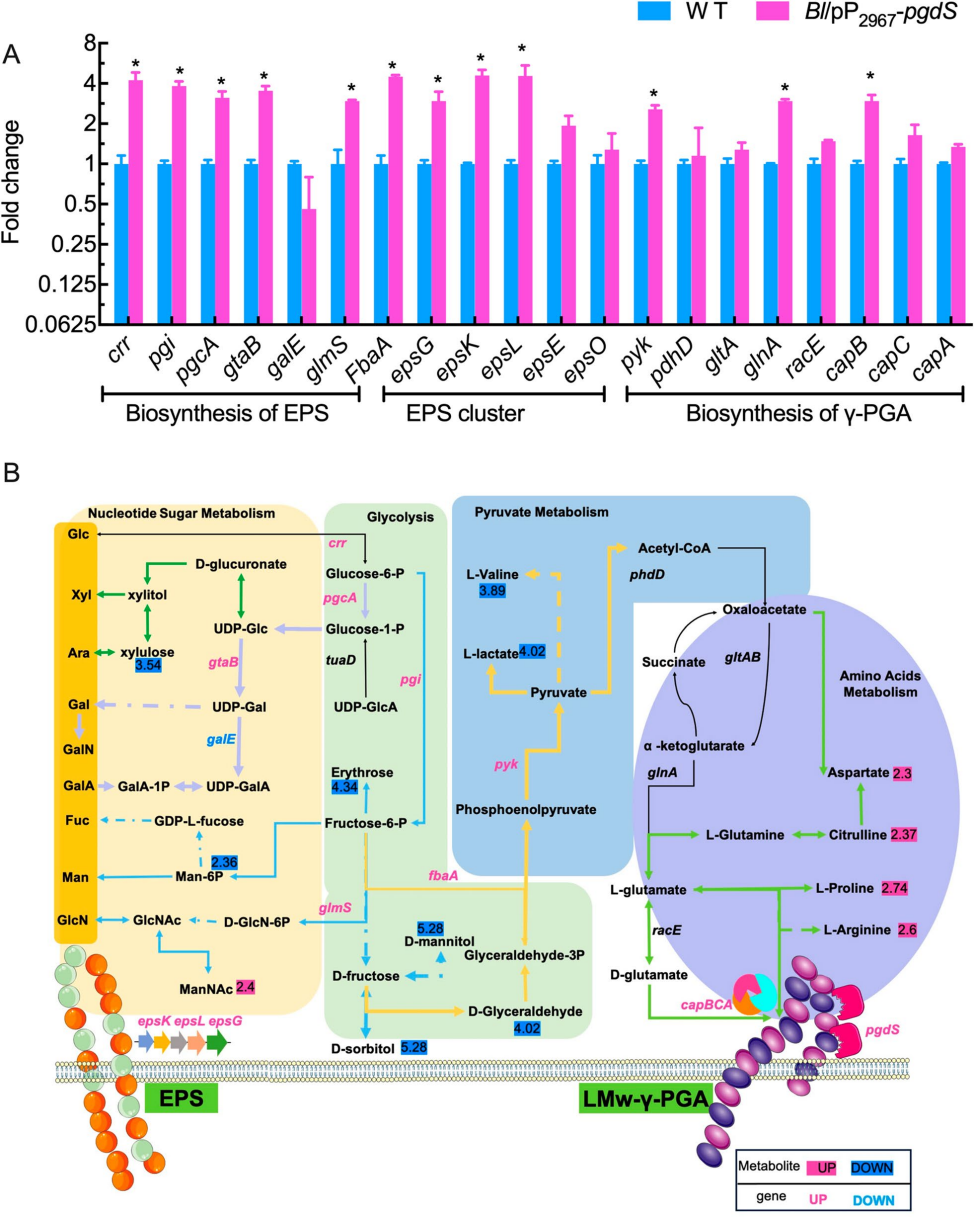
The specific metabolites and genes in the main metabolic pathways in wild-type and *Bl/*pP_2967_*-pgdS* strains. **A** Analysis of gene expression in the EPS and γ-PGA synthesis pathway. **B** Analysis of metabolites in the main metabolic pathways. Red: increase, Blue: decrease, *: *p* < 0.05

For a comprehensive understanding of the mechanism underlying EPS and γ-PGA improvement in *pgdS* engineered strains, transcriptional analysis was performed using RT-qPCR to compare the related gene expression levels of the wild-type strain and *Bl/pP*_2967_*-pgdS* (Fig. 5A). The differentially abundant metabolites and several key genes were further mapped to the major metabolic pathways (Fig. 5B), including glycolysis, the TCA cycle, amino acid metabolism, γ-PGA, and EPS biosynthesis.

Most metabolites in the branching of the EPS synthesis pathway were decreased, while most metabolites in the amino acid metabolism of the γ-PGA synthesis pathway were upregulated in *Bl*/pP_2967_-*pgdS*. The content of xylose (Xyl) only existed in *Bl*/pP_2967_-*pgdS* and not in the wild-type strain.

There are two carbon flow branches downstream of glucose 6-phosphate, fructose 6-phosphate, and glucose 1-phosphate. Glucose 1-phosphate was the precursor for the synthesis of erythrose, and the yield of erythrose decreased by 4.34 times, suggesting that more carbon flow went to the pathway of glucose 1-phosphate. Glucose 1-phosphate is divided into UDP-Glc and UDP-Gal downstream. Xylulose involved in nucleotide sugar metabolism was decreased, which was the precursor substance for arabinose (Ara), and presumably more carbon flow into xylose synthesis. Subsequently, during the biosynthesis of GalN and Gal, the expression of the glucose-phosphate transferase gene *crr*, glucose-phosphate mutase gene *pgcA,* and UDP-glucose pyrophosphorylase gene *gtaB* was upregulated, while the UDP-glucose 4-isomerase gene *galE* was downregulated, which is involved in the biosynthesis of GalA. It was speculated that more carbon flow enriched Gal and GalN synthesized downstream of UDP-Gal. Metabolites involved in the synthesis of sugar alcohols (erythritose, sorbitol, and mannitol) were decreased, and the carbon flux of GlcN and Fuc was presumed to increase. The 2.36-fold decrease in Man-6P suggests that the carbon flux may shift from Man to Fuc synthesis. GlcN also increased due to the increased supply of *N*-acetyl-d-mannosamine (ManNAc). The expression levels of EPS synthesis cluster genes were increased in *Bl/pP*_2967_*-pgdS*. In particular, there was a 4.0- to 4.6-fold increase in the expression of *epsK*, *epsL*, and *epsG*, which are responsible for the transport of polysaccharide repeating units [33]. Additionally, during γ-PGA production, the glutamate synthase gene *gltA* and γ-PGA synthase gene *capB* were significantly upregulated. The arginine and proline in *Bl/pP*_2967_*-pgdS* were upregulated by 2.6- and 2.7-fold, respectively, which can be converted to glutamate and provide substrate for the synthesis of γ-PGA. Citrulline and aspartic acid were upregulated 2.4- and 2.3-fold, respectively, which provided a precursor for the TCA cycle and glutamate and generated ATP [34].

The increased expression of *pgdS* in *Bl/pP*_2967_*-pgdS* is speculated to be responsible for the improvement of EPS and γ-PGA. The degradation of γ-PGA is caused by PgdS hydrolase, which is an endo-γ-glutamyl peptidase of the NlpC/P60 family. PgdS carries three NlpC/P60 domains, of which the core catalytic domain contains the complete triad cysteine/histidine/glutamine (Cys194-His247-Gln259) [35]. Around the catalytic cysteine, aspartic acid, serine, and tyrosine are strictly conserved in domain 2 of PgdS (corresponding residues Asp193, Ser195, and Tyr181), which are associated with substrate-specific binding, and domains 1 and 3 have proline and threonine residues, respectively [36]. In *Bl/pP*_2967_*-pgdS*, proline and aspartic acid can be converted to glutamic acid to provide substrates for γ-PGA synthesis. On the other hand, it can also improve the ability of specific binding of the PgdS enzyme to substrates, and thus, PgdS catalyses more substrates. In addition to PgdS, which is strictly dedicated to γ-PGA hydrolysis, other D/L endopeptidases (LytE, LytF, Cwl, Cwlo, and Cwlt) have also been shown to hydrolyse γ-PGA [37]. Furthermore, with the increase in *pgdS* expression, the degradation efficiency of γ-PGA was significantly improved, and γ-PGA with a low molecular weight was obtained, which affected the synthesis of extracellular polymeric substances. We speculated that low-Mw γ-PGAs lead to a more polydisperse extracellular polymeric substance product, and the EPS attached to the membrane will have more space to transport from intracellular to extracellular, improving the transportation efficiency of EPS and further increasing the EPS yield. The expression levels of polysaccharide transporters (*epsK*, *epsL*, and *epsG*) showed a significant increase (4.0- to 4.6-fold) in *Bl*/pP_2967_-*pgdS*. Thus, enhanced amino acid metabolism stimulates substrate transfer between the PgdS enzyme and γ-PGA, and low-Mw γ-PGA provides a low viscosity and loose extracellular space, which is conducive to EPS transport.

### Scale-up of LMw-γ-PGA production on 1.5-L fermentor

**Fig 6:**
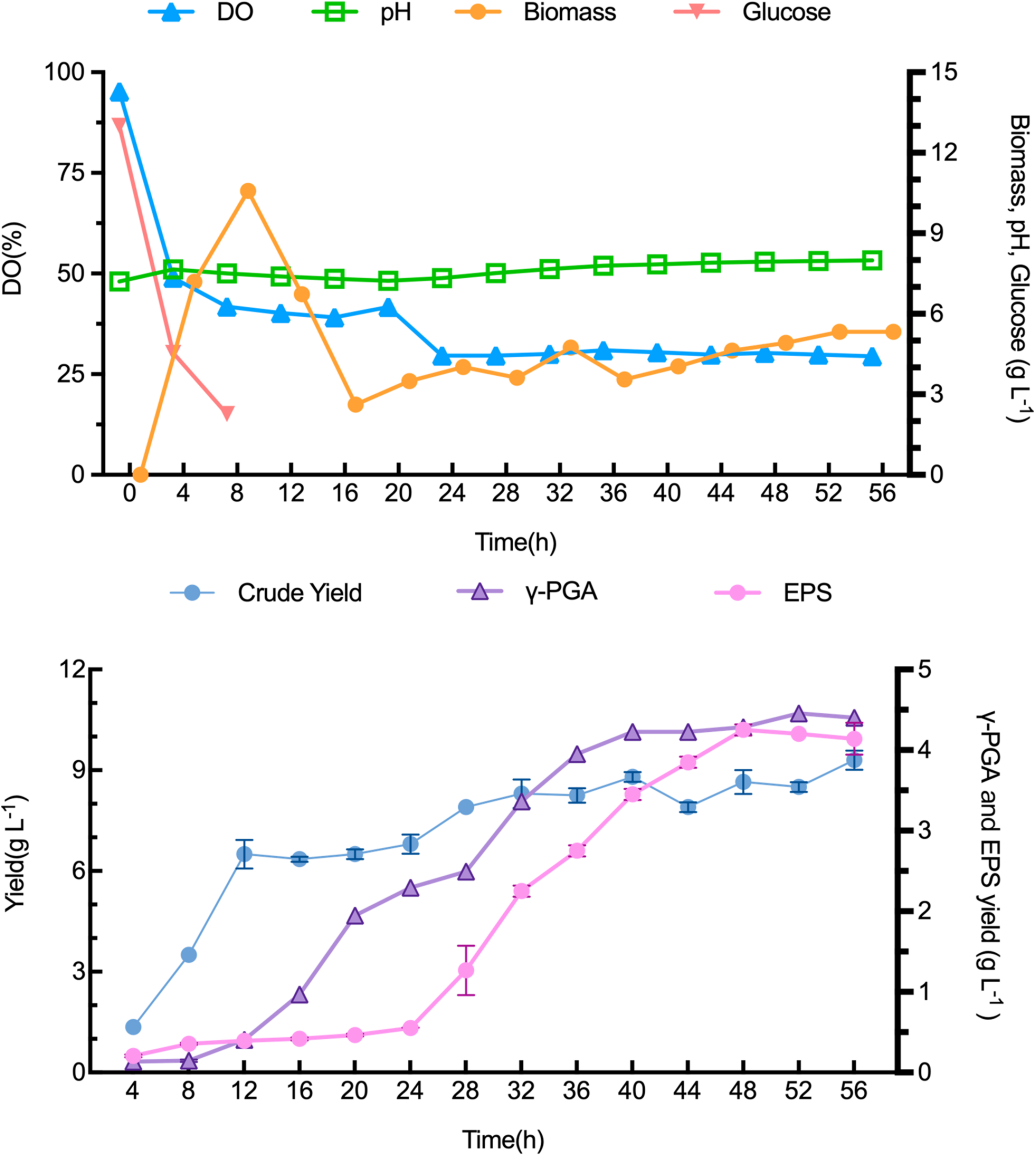
The fermentation process curve of strain *Bl*/pP_2967_*-pgdS* on 1.5-L fermentor

The ability of *Bl*/pP_2967_-*pgdS* to produce EPS and LMw-γ-PGA during large-scale fermentation was investigated on a 1.5-L fermentor. *B. licheniformis* CGMCC 2876 was also fermented in another 1.5-L fermentor as a control strain (Additional file 1: Fig. S6). A typical fermentation profile in terms of DO, pH, biomass, and γ-PGA and EPS production is shown in Fig. 6. The rise in pH is believed to be caused by the heightened synthesis of extracellular polymeric substances, which creates a favourable alkaline milieu for the production of γ-PGA. Following inoculation, the concentration of dissolved oxygen gradually declined. Concurrently, the remaining glucose content in the medium was swiftly depleted, with a carbon source consumption rate of 0.56 g/h·L. Subsequently, at 4 h, a substantial surge in biomass was observed, indicating rapid bacterial proliferation. The Mw of γ-PGA produced by *Bl*/pP_2967_-*pgdS* remained stable at 1.6 × 10^3^ kDa at the peak time of 20 min, which was later than that of the wild-type strain (16 min) (Additional file 1: Fig. S5). Cell growth reached a stable stage after 24 h, followed by a gradual increase in the production of extracellular polymeric substances, suggesting a positive correlation between extracellular polymeric substance production and cell growth. After 56 h of fermentation, the yield of extracellular polymeric substances reached 9.3 g/L by *Bl*/pP_2967_-*pgdS*, with a 10.2-fold increase in EPS yield and a 29.4% increase in γ-PGA yield compared to the wild-type strain. As a result, the EPS and γ-PGA production of *Bl*/pP_2967_-*pgdS* reached 4.2 g/L and 4.4 g/L, respectively (Fig. 6B).

## Discussions

The Mw of γ-PGA varies with producing strains and culture conditions. In addition to traditional methods such as strain screening and culture-medium optimization, more efforts are being made to manipulate γ-PGA synthetase and γ-PGA hydrolase using genetic engineering techniques for constructing γ-PGA production strains with specific Mws. Remarkably, the promoter is the switch that controls the expression of target genes, and promoter engineering has been proven to be an effective strategy for executing accurate coordination in metabolite production [38]. Nevertheless, the available promoters for *B. licheniformis* are limited [39] and are mostly universal promoters from *B. subtilis* and others [40]; little is known about endogenous promoters in *B. licheniformis.* Among the constructed gradient endogenous promoters, the P_2640_, P_2967_, and P_3232_ promoters gave strong green fluorescence intensities and increased *pgdS* expression levels. In particular, the average Mw of γ-PGA from *Bl*/pP_2967_-*pgdS* was 1.61 × 10^3^ kDa, which was the largest reduction in Mw. Furthermore, sequence alignment revealed that P_2967_ was the promoter belonging to acetolactate synthase AlsS*,* which is a key enzyme in the synthesis of branched amino acids such as valine (Val), leucine (Leu), and isoleucine (Ile) in microorganisms [41].

This study illustrates a promising approach for the improvement of extracellular polymeric substances with a low Mw of γ-PGA in one strain through the expression levels of *pgdS* using manipulation of the endogenous promoters from *B. licheniformis* CGMCC 2876. The viscosity is an important factor for γ-PGA production, and influenced by γ-PGA Mw [42, 43]. The *pgdS* engineered strain *Bl/*pP_2967_*-pgdS* with the lowest γ-PGA Mw increased extracellular polymeric substance production by 12.8% with low viscosity, which may be the low viscosity favouring oxygen transfer and substrate utilization [44]. Compared with the wild-type strain, the *Bl*/pP_3232_-*pgdS* and *Bl/pP*_2640_-*pgdS* strains showed increased γ-PGA production of 11.3% and 12.9%, respectively, with low viscosity (87 and 90 mPa.s). Similarly, Tian et al. reported that γ-PGA yield reached 20.16 g/L in *B. licheniformis* WX-02 with enhanced expression of *pgdS* using the P43 promoter, an increase of 54% over that of the control strain (13.11 g/L), and the expression level of the *pgdS* gene was almost 10 times higher than that of the control strain [18]. The production of LMw-γ-PGA (6.82 × 10^4^ Da) in the *pgdS* recombinant strain with P43 and the signal peptide SPsacB in combination increased 34.89% compared with *B. licheniformis* WX-02 [19]. Remarkably, both EPS (9.9-fold) and γ-PGA (16.54%) production increased in *Bl*/pP_2967_-*pgdS*. Our study indicated that overexpression of *pgdS* is an efficient approach to simultaneously enhance γ-PGA and EPS production.

Subsequently, EPS synthesis in *pgdS* engineered strains changed greatly with decreasing Mw. The γ-PGA Mw of *Bl*/pP_3388_-*pgdS*, *Bl*/pP_3937_-*pgdS,* and *Bl*/pP_2967_-*pgdS* decreased to 4.99 × 10^3^, 4.39 × 10^3^ and 1.61 × 10^3^ kDa, respectively, and these strains produced more than 4 g/L EPS. We speculated that low Mw γ-PGAs lead to a more polydisperse extracellular polymeric substance product, and the EPS attached to the membrane will have more space to transport from the intracellular to extracellular space, improving the transportation efficiency of EPS and further increasing the EPS yield. The decrease in viscosity (93 mPa.s) from *Bl/*pP_2967_*-pgdS* led to an increase in the production of EPS (4.24 g/L). Along with EPS, the biosynthesis of EPS also requires a high concentration of transport protein. The expression levels of polysaccharide transporters (*epsK*, *epsL*, and *epsG*) showed a significant increase (4.0- to 4.6-fold) in *Bl*/pP_2967_-*pgdS*. Other studies found that EpsK contains the conserved domain Wzx, and the protein was postulated to be a flippase or glycosyltransferase for the transportation of polysaccharide repeating units from the cytoplasm to the periplasmic side [45]. EpsL may be a glucosyl or galactosyltransferase, and EpsG was involved in the transport of 3-galactosyltransferase [46]. Combined with the increased components of EPS, LMw-γ-PGA created more space to improve the transportation efficiency of EPS, with increased expression levels of *epsK*, *epsH,* and *epsL*, which may be involved in the transport of galactose, galactosamine hydrochloride, and glucosamine hydrochloride.

EPS compositions depend on strains, synthesis genes, and sugar substrate [47]. *Bacillus sp*. can use carbohydrates as the energy source for its own metabolism [48, 49]. Here, the observed reduction in neutral sugars might be due to consumption in *Bl*/pP_2967_-*pgdS*, similar to glucose. Moreover, the content of Gal, the component with the largest proportion of neutral sugars, increased significantly in *Bl*/pP_2967_-*pgdS,* with enrichment of galactosamine hydrochloride (GalN). Galacturonic acid was decreased from 3.67 mol% to 0 mol% due to the downregulation of the related gene of *galE*, which encodes UDP-galactose-4-epimerase. These metabolites involved in the UDP-Gal pathway changed the carbon flow to enrich the synthesis of Gal and GalN. In addition, the contents of GlcN and Fuc accumulated, whereas the metabolites involved in glycoalcohol synthesis (erythrose, sorbitol, and mannitol) were reduced. This result suggested that the carbon flux downstream of fructose-6-P could be redirected away from Man towards Fuc synthesis. Moreover, the expression level of genes encoding glutamine fructose-6-phosphate transaminase gene *glmS* was also upregulated. GlcN, as an amino sugar, also increased due to the enhanced *N*-acetyl-d-mannosamine (ManNAc) supply. Similar results were reported in which an enhanced ManNAc supply could improve carbohydrate production at the outer surface of glycans [50]. These results might suggest that fucose, galactose, glucosamine hydrochloride, and xylose accumulation is related to the increased carbon flow in *Bl*/pP_2967_-*pgdS*.

The γ-PGA produced by *B. licheniformis* CGMCC 2876 has a Mw of 1.25 × 10^5^ kDa, which is a high MW γ-PGA and can be used as a flocculant [8]. The *pgdS* engineered strains with different endogenous promoters can obtain low-Mw γ-PGA, which has good application prospects in agriculture and the pharmaceutical industries [4]. For example, γ-PGA has biocontrol capacity and antimicrobial activities, it can be applied to protect seedlings from the adverse effects [51]. Moreover, Mw of γ-PGA was the decisive factor for controlling or delaying the drug delivery release, which can be suitable carriers for gene therapy [52]. Although LMw-γ-PGA was obtained in this study, the range of Mws (1.61 × 10^3^ to 2.03 × 10^4^ kDa) was still narrow compared with those reported by Sha et al. (< 1500 kD) [20] and Halmschlag et al. (40–8500 kDa) [2], which could be due to the specific γ-PGA synthetase of the native strain. Furthermore, the yield of low Mw (1.61 × 10^3^ kDa) γ-PGA (4.4 g/L) can be obtained by a 1.5-L fermentor in *Bl*/pP_2967_-*pgdS*. The EPS yield was 10.2-fold higher than that of the wild-type strain, and the γ-PGA yield was increased by 29.4%. Therefore, low Mw γ-PGA can promote the synthesis of extracellular polymeric substances, especially substantially increased EPS, which has a positive effect on exploring the mechanism of the synthesis of EPS and γ-PGA and provides a strategy for promoting the synthesis of extracellular polymeric substances.

## Conclusion

In this study, the expression of γ-PGA-degrading enzyme (PgdS) was regulated by various promoters, producing γ-PGAs with specific Mws ranging from 1.61 × 10^3^ to 2.03 × 10^4^ kDa. More interestingly, EPS production increased markedly in *Bl*/pP_2967_-*pgdS* with lower Mw and viscosity. The enhanced carbon flow of galactose, galactosamine, and glucosamine hydrochloride increased the expression of polysaccharide transporters (*epsK*, *epsL,* and *epsG*) for EPS synthesis in this strain. It was suggested that the enhanced regulation of *pgdS* decreased the Mw of γ-PGA, thereby promoting the production of EPS and γ-PGA. This study provides deep insight into the synthesis mechanism of EPS and γ-PGA and provided an effective strategy for the controlled synthesis of extracellular polymeric substances.

## Materials and methods

### Strains and cultivation

*Escherichia coli* strain DH5α was used as the host for cloning. The constructed *B. licheniformis* strains used and associated plasmids in this study are presented in Additional file 1: Table S1 and Table S2. *B. licheniformis* was primarily cultured in seed medium and transferred to fermentation medium as a 4% inoculum. One litre of fermentation medium contained 13.9 g glucose, 0.048 g MgSO_4_, 5.6 g KH_2_PO_4_, 1.4 g K_2_HPO_4_, 2 g NaCl, 2.67 g urea, and 0.6 g yeast extract [21]. When necessary, antibiotics (10 or 50 μg/L tetracycline) were supplemented into the media. The pH of the seed and fermentation media was adjusted to 7.2. All strains were precultured for cell growth in Luria–Bertani (LB) medium containing 10 g/L NaCl, 5 g/L yeast extract, and 10 g/L tryptone [53]. All strains were precultured in LB solid medium with proper antibiotics at 37 °C overnight. Strains from single colonies on plates were inoculated in fresh seed medium for 18 h at 200 rpm. Four per cent (v/v) overnight seed cultures of strains were inoculated in fresh EPS medium containing appropriate antibiotics with an initial OD_600_ of 1.8–2.0 (middle logarithmic period of growth). After cultivation at 37 °C and 200 rpm for 56 h, samples were collected for analysis.

### Prediction of endogenous promoter and construction of recombinant strains of GFP

In an earlier project, the transcriptome of *B. licheniformis* was selected for sequencing. Then, we screened for endogenous constituent promoters based on transcription strength (Additional file 1: Table S3). The core regions of these promoters were predicted by http://linux1.softberry.com/berry.phtml?topic=bprom&group=programs&subgroup=gfindb.

The green fluorescent protein reporter gene GFP expression plasmids were constructed based on gene overexpression plasmids pHY300PLK-P*amyL*-TT*amyL*, according to our previously reported method [33]. The promoter DNA region complex of 8 promoters was amplified from the genomic DNA of *B. licheniformis* CGMCC 2876. The RBS was attained through PylB (5′-GAAACAACAAAGGGGGAGATTTGT-3′) [27]. The construction procedure of the GFP expression vector pP_3232_-GFP mediated by promoter P_3232_ served as an example. Briefly, the P_3232_ promoter, RBS, and GFP genes were fused by splicing overlap extension (SOE)-PCR to obtain the expression cassettes. The amplified fragment had *Hind* III and *Kpn*I sites 5′ and 3′, respectively. The expression cassettes were inserted into *Hind* III/*Kpn*I-cut pHY300PLK-P*amyL*-TT*amyL*, and the ligation product was used to transform *E. coli* DH5α, yielding the plasmid pHY-P_3232_-GFP. The resulting plasmids were transformed into *B. licheniformis* CGMCC 2876 to obtain the recombinant strain *Bl*/pP_3232_-GFP. Moreover, the control strain was constructed without a promoter before the GFP gene. The sequences of the primers used in this study are listed in Additional file 1: Table S5.

### Construction of recombinant strains of* pgdS*

A series of *pgdS* expression vectors were constructed by a similar method. As an example, the construction procedure of the plasmid pP_2640_-*pgdS* (containing the P_2640_ promoter and *pgdS* gene) was described. First, the *pgdS* gene of *B. licheniformis* CGMCC 2876 was amplified. Then, the resulting fragment was cloned and inserted into the plasmid pHY-P_2640_-GFP to replace the GFP gene using the BamHI and KpnI sites, generating pHY-P_2640_-*pgdS*. Then, these gene overexpression vectors were transferred into *B. licheniformis* by electroporation, resulting in the gene overexpression strain *Bl*/pP_2640_-*pgdS*. Notably, all the recombinant vectors were verified by DNA sequencing. All colonies were selected and characterized.

### Scale-up on 1.5-L fermentor

For scale-up production on a 1.5-L fermentor (CloudReady™ parallel-bioreactor, made in China), *B. licheniformis* was cultivated in 0.9 L fermentation medium. For scale-up fermentation, *B. licheniformis* was primarily cultured in seed medium and transferred to fermentation medium. When necessary, antibiotics (10 or 50 μg/L tetracycline) were supplemented into the media of the engineered strain. The inoculation ratio of fermentation medium was 4% seed culture, and the aeration rate was 2.0 vvm throughout the fermentation process. The culture was carried out at 37 °C, and the dissolved oxygen (DO) was associated with stirring to maintain the dissolved oxygen no lower than 30%. Samples were taken every 4 h and then subjected to further analysis.

### Analytical methods

The relative fluorescence intensity of the GFP-expressing recombinants was measured by fluorometry [28]. Then, 96-well black plates (Corning, USA) were subjected to relative fluorescence intensity and cell optical density determination using a Multi-Mode Microplate Reader (SpectraMax iD3; Molecular Devices). An excitation wavelength of 480 nm and an emission wavelength of 516 nm were used to determine the relative fluorescence intensity of GFP. The cell density was measured at a wavelength of 600 nm. The samples were tested in triplicate. Single-cell fluorescence was viewed with a fluorescence microscope (DM2500, Leica). The cells were cultured for 24 h, washed twice in phosphate-buffered saline, and diluted 1:10 with phosphate-buffered saline. The dynamic viscosity of fermentation broth was measured using a viscometer at 25 °C (NDJ-8 S, Shanghai, China) in triplicate. The differences in morphology and extracellular polymeric substances were evaluated using SEM (Sigma, Hitachi, Tokyo, Japan).

After the fermentation process, the biopolymer was extracted and purified using the method described in a previous study [54]. For γ-PGA content, 10 mL of cell culture was centrifuged at 10,000 rpm for 5 min, digested with hydrochloric acid, and neutralized to pH 7. The samples were filtered through 0.22 µm PVDF membrane syringe filters (Whatman Inc.). The γ-PGA yields were determined by high-performance liquid chromatography (HPLC, Shimadzu Prominence HPLC System equipped with UV detector SPD-20AV) [54]. In addition, 20 μl of filtered sample was injected into an HC-C_18_ column (250 × 4.6 mm, Agilent Technologies, Santa Clara, CA, USA) and eluted by solvent A (95% 0.1 M KH_2_PO_4_) and solvent B (5% methanol). The total flow rate was maintained at 1 mL/min. The column temperature was maintained at 30 °C, and the UV detection wavelength was 210 nm. The weight average molecular weight of γ-PGA was measured using HPSEC. The samples were filtered through a 0.22-μm nylon syringe filter. Twenty microlitres of sample was injected into an Agilent HPLC system equipped with an RID detector. HPLC conditions: TSKgel GMPWXL (7.8 × 300 mm, 13 μm, Tosoh), mobile phase: water, flow rate: 0.3 mL/min, column temperature: 35 °C, time: 65 min. The glucan standards of 2000 kD, 500 kD, 200 kD, 70 kD, 40 kD, and 10 kDa (Shanghai Aladdin Biotechnology Co., Ltd., Shanghai, China) were employed to establish a calibration curve. The content of total carbohydrates in the biopolymer was detected using the phenol–sulfuric acid method [33]. Reducing sugar concentrations were determined by the 3,5-dinitrosalicylic acid (DNS) method. The cell density (OD_600_) was measured using a UV-1800 spectrophotometer (Shimadzu Global Laboratory Consumables Co., Ltd., Shanghai, China). Moreover, high-performance ion exchange chromatography (HPIEC) was employed to analyse the monosaccharide composition of the extracellular polymer [55]. Monosaccharides, including fucose (Fuc), rhamnose (Rha), arabinose (Ara), glucosamine hydrochloride (GlcN), galactose (Gal), glucose (Glc), *N*-acetyl-d-glucosamine (GlcNA), xylose (Xyl), mannose (Man), fructose (Fru), ribose (Rib), galacturonic acid (GalA), glucuronic acid (GlcA), D-galactosamine hydrochloride (GalN), guluronic acid (GulA), and mannose acid (ManA), were used as analytical standards.

### Real-time quantitative PCR analysis (RT-qPCR)

When the cells grew into the mid-logarithmic growth phase, the cells were collected for RNA extraction according to Xu’s method [33]. HiScript®II Q RT SuperMix for qPCR (+ gDNA wiper) (Vazyme, China) was employed for cDNA synthesis. RT-qPCR was performed using iTaqTM Universal SYBR® Green Supermix (Bio-Rad, United States). The experiments were performed in three replicates, and 16S rRNA was used as the reference gene [56]. The relative transcriptional level of genes was calculated using the 2^–ΔΔCt^ method.

### Metabolomics analysis

When the cells were grown for 24 h, the culture medium was removed by centrifugation. The cell suspension was washed three times with 10 mM PBS. Cells were resuspended in 1 mL PBS and centrifuged at 4 °C. Cells were precipitated in liquid nitrogen for 1 min. Then, 1 mL of MeOH:ACN:H_2_O (V:V:V, 2:2:1) was vortexed for 30 s for the following 10 min sonication (4 °C water bath). The samples were placed in liquid nitrogen for 1 min. This procedure was repeated 3 times to burst the cells in total. Then, the samples were incubated at − 20 °C for 1 h to facilitate protein precipitation. Finally, the samples were freeze-centrifuged (1300 rpm, 15 min), and the supernatant was collected for freeze-drying by an FDU-1200 freeze-drying machine. The resulting sample was analysed on a TripleTOF 5600 + Liquid chromatograph-mass spectrometer (LC–MS). Five replicates were used in this experiment. Metabolomics data have been deposited in the iProX database (www.iprox.cn) with accession number IPX0006376000.

### Statistical analysis

SIMCA software and MetaboAnalyst (https://www.metaboanalyst.ca/) were used to screen different metabolites. The data are represented as the mean ± SD. SPSS software 20 (IBM, New York, USA) was used to perform one-way analysis of variance and *t* test significance analysis. *p* < 0.05 indicated significance. GraphPad Prism 9.0 software (GraphPad Software, CA, USA) was used to draw figures.

### Supplementary Information

**Additional file 1: Table S1**. Plasmids used in this study. **Table S2**. Strains used in this study. **Table S3.** Transcriptome data of different promoters used in this study. **Table S4**. The promoters used in this study. **Table S5.** Primers used for PCR in this study. Table S6 Identification of differential metabolites of wild-type and *Bl*/pP_2967_-*pgdS* strains. **Fig S1.** The transcriptional levels of gene *pgdS* among strains with different promoters. **Fig S2.** A heatmap of the pearson's correlation coefficients produced by comparing metabolites significantly affected by wild-type and *pgdS* engineered strains. Positive correlations are shown in red; negative correlations are shown in blue. **Fig. S3.** Permutation test of cross-validation (200 permutations) for the OPLS-DA model in wild-type and *Bl*/pP2967-pgdS strains. **Fig S4.** The KEGG enrichment analysis of different metabolites screened in positive (A) and negative (B) ion mode. **Fig S5.** Molecular weight analysis of γ-PGA between wild-type and *Bl*/pP2967-pgdS strains. **Fig S6.** The synthesis products of *B. licheniformis* CGMCC 2876 in 1.5-L fermentor.

## Data Availability

The data sets generated or analyzed during this study are included in this published article and its supplementary materials.
