# Supplementary material for: Molecular weight control of poly-γ-glutamic acid reveals novel insights into extracellular polymeric substance synthesis in Bacillus licheniformis
Source: Biotechnol Biofuels Bioprod. 2024 May 6;17:60. doi: 10.1186/s13068-024-02501-9 (PMC11075323; doi:10.1186/s13068-024-02501-9)
Supplement: Supplementary file 1 — Additional file 1: Table S1. Plasmids used in this study. Table S2. Strains used in this study. Table S3. Transcriptome data of different promoters used in this study. Table S4. The promoters used in this study. Table S5. Primers used for PCR in this study. Table S6 Identification of differential metabolites of wild-type and Bl/pP2967-pgdS strains. Fig S1. The transcriptional levels of gene pgdS among strains with different promoters. Fig S2. A heatmap of the pearson's correlation coefficients produced by comparing metabolites significantly affected by wild-type and pgdS engineered strains. Positive correlations are shown in red; negative correlations are shown in blue. Fig. S3. Permutation test of cross-validation (200 permutations) for the OPLS-DA model in wild-type and Bl/pP2967-pgdS strains. Fig S4. The KEGG enrichment analysis of different metabolites screened in positive (A) and negative (B) ion mode. Fig S5. Molecular weight analysis of γ-PGA between wild-type and Bl/pP2967-pgdS strains. Fig S6. The synthesis products of B. licheniformis CGMCC 2876 in 1.5-L fermentor. [file 13068_2024_2501_MOESM1_ESM.docx]

**Supplemental material**

**Molecular weight control of poly-γ-glutamic acid reveals novel insights into extracellular polymeric substances synthesis in *Bacillus licheniformis***

**Xiaoyu Wei^a,b^, Lijie Yang^a,b^, Zhen Chen^c*^, Wenhao Xia^a,b^, Yongbin Chen^a,b^, Mingfeng Cao^a,b^*, Ning He^a,b^***

^a^ Department of Chemical and Biochemical Engineering, College of Chemistry and Chemical Engineering, Xiamen University, Xiamen, Fujian 361005, China

^b^ The Key Lab for Synthetic Biotechnology of Xiamen City, Xiamen University, Xiamen, People’s Republic of China

^c^ College of Life Science, Xinyang Normal University, Xinyang 464000, China.

*Corresponding Author:

**Ning He**: Department of Chemical and Biochemical Engineering, College of Chemistry and Chemical Engineering, Xiamen University, Xiamen, Fujian 361005, China

Email: [hening@xmu.edu.cn](mailto:hening@xmu.edu.cn)

**Mingfeng Cao**: Department of Chemical and Biochemical Engineering, College of Chemistry and Chemical Engineering, Xiamen University, Xiamen, Fujian 361005, China

Email: [mfcao@xmu.edu.cn](mailto:mfcao@xmu.edu.cn)

**Zhen Chen**: College of Life Science, Xinyang Normal University, Xinyang 464000, China.

E-mail: chenzhen@xynu.edu.cn

**Table S1**. Plasmids used in this study.

| Plasmid | Description | Source |
| --- | --- | --- |
| pHY300PLK | *E. coli*-*Bacillus* shuttle vector; Amp^R^ in *E. coli*,  Tc^R^ in both *E. coli* and *B. subtilis* | Stored in this lab |
| pHY-sfGFP | pHY300PLK derivative carrying superfolder GFP gene | This study |
| pHY-P_3515_-GFP | pHY300PLK derivative carrying P_3515_ promoter, GFP gene | This study |
| pHY-P_3232_-GFP | pHY300PLK derivative carrying P_3232_ promoter, GFP gene | This study |
| pHY-P_2640_-GFP | pHY300PLK derivative carrying P_2640_ promoter, GFP gene | This study |
| pHY-P_2967_-GFP | pHY300PLK derivative carrying P_2967_ promoter, GFP gene | This study |
| pHY-P_2097_-GFP | pHY300PLK derivative carrying P_2097_ promoter, GFP gene | This study |
| pHY-P_3388_-GFP | pHY300PLK derivative carrying P_3388_ promoter, GFP gene | This study |
| pHY-P_3588_-GFP | pHY300PLK derivative carrying P_3588_ promoter, GFP gene | This study |
| pHY-P_3937_-GFP | pHY300PLK derivative carrying P_3937_ promoter, GFP gene | This study |
| pHY-P43-GFP | pHY300PLK derivative carrying P43 promoter, GFP gene | This study |
| pHY-P_spoVG_-GFP | pHY300PLK derivative carrying Pspo promoter, GFP gene | This study |
| pHY-P_3232_-*pgds* | pHY300PLK derivative carrying P_3232_ promoter, pgdS gene | This study |
| pHY-P_2640_-*pgds* | pHY300PLK derivative carrying P_2640_ promoter, pgdS gene | This study |
| pHY-P_2967_-*pgds* | pHY300PLK derivative carrying P_2967_ promoter, pgdS gene | This study |
| pHY-P_2097_-*pgds* | pHY300PLK derivative carrying P_2097_ promoter, pgdS gene | This study |
| pHY-P_3388_-*pgds* | pHY300PLK derivative carrying P_3388_ promoter, pgdS gene | This study |
| pHY-P_3588_-*pgds* | pHY300PLK derivative carrying P_3588_ promoter, pgdS gene | This study |
| pHY-P_3937_-*pgds* | pHY300PLK derivative carrying P_3937_ promoter, pgdS gene | This study |
| pHY-P_spoVG_-*pgds* | pHY300PLK derivative carrying Pspo promoter, pgdS gene | This study |
| pHY-P43-*pgds* | pHY300PLK derivative carrying P43 promoter, pgdS gene | This study |

**Table S2**. Strains used in this study.

| Strain | Description | Source |
| --- | --- | --- |
| DH5α | *E. coli* |  |
| DH5α/p-GFP | DH5α contains plasmid pHY-GFP | This study |
| DH5α/pP_3515_-GFP | DH5α contains plasmid pHY-P_3515_-GFP | This study |
| DH5α/pP_3232_-GFP | DH5α contains plasmid pHY-P_3232_-GFP | This study |
| DH5α/pP_2640_-GFP | DH5α contains plasmid pHY- P_2640_-GFP | This study |
| DH5α/pP_2967_-GFP | DH5α contains plasmid pHY-P_2967_-GFP | This study |
| DH5α/pP_2097_-GFP | DH5α contains plasmid pHY-P_2097_-GFP | This study |
| DH5α/pP_3388_-GFP | DH5α contains plasmid pHY-P_3388_-GFP | This study |
| DH5α/pP_3588_-GFP | DH5α contains plasmid pHY-P_3588_-GFP | This study |
| DH5α/pP_3937_-GFP | DH5α contains plasmid pHY-P_3937_-GFP | This study |
| DH5α/pP43-GFP | DH5α contains plasmid pHY-P43-GFP | This study |
| DH5α/pP_spoVG_-GFP | DH5α contains plasmid pHY-P_spo_-GFP | This study |
| DH5α/pP_3232_-*pgds* | DH5α contains plasmid pHY-P_3232_-*pgds* | This study |
| DH5α/pP_2640_-*pgds* | DH5α contains plasmid pHY- P_2640_-*pgds* | This study |
| DH5α/pP_2967_-*pgds* | DH5α contains plasmid pHY-P_2967_-*pgds* | This study |
| DH5α/pP_2097_-*pgds* | DH5α contains plasmid pHY-P_2097_-*pgds* | This study |
| DH5α/pP_3388_-*pgds* | DH5α contains plasmid pHY-P_3388_-*pgds* | This study |
| DH5α/pP_3588_-*pgds* | DH5α contains plasmid pHY-P_3588_-*pgds* | This study |
| DH5α/pP_3937_-*pgds* | DH5α contains plasmid pHY-P_3937_-*pgds* | This study |
| DH5α/pP43-*pgds* | DH5α contains plasmid pHY-P43-*pgds* | This study |
| DH5α/pP_spoVG_-*pgds* | DH5α contains plasmid pHY-P_spo_-*pgds* | This study |
| *Bacillus licheniformis* CGMCC 2876 | Wild-type strain | Stored in lab |
| *Bl*/p-GFP | *B. licheniformis* CGMCC 2876 contains plasmid pHY-GFP | This study |
| *Bl*/pP_3515_-GFP | *B. licheniformis* CGMCC 2876 contains plasmid pHY-P_3515-_GFP | This study |
| *Bl*/pP_3232_-GFP | *B. licheniformis* CGMCC 2876 contains plasmid pHY-P_3232_-GFP | This study |
| *Bl*/pP_2640_-GFP | *B. licheniformis* CGMCC 2876 contains plasmid pHY- P_2640_-GFP | This study |
| *Bl*/pP_2967_-GFP | *B. licheniformis* CGMCC 2876 contains plasmid pHY-P_2967_-GFP | This study |
| *Bl*/pP_2097_-GFP | *B. licheniformis* CGMCC 2876 contains plasmid pHY-P_2097_-GFP | This study |
| *Bl*/pP_3388_-GFP | *B. licheniformis* CGMCC 2876 contains plasmid pHY-P_3388_-GFP | This study |
| *Bl*/pP_3588_-GFP | *B. licheniformis* CGMCC 2876 contains plasmid pHY-P_3588_-GFP | This study |
| *Bl*/pP_3937_-GFP | *B. licheniformis* CGMCC 2876 contains plasmid pHY-P_3937_-GFP | This study |
| *Bl*/pP43-GFP | *B. licheniformis* CGMCC 2876 contains plasmid pHY-P43-GFP | This study |
| *Bl*/pP_spoVG_-GFP | *B. licheniformis* CGMCC 2876 contains plasmid pHY-P_spo_-GFP | This study |
| *Bl*/pP_3232_-*pgds* | *B. licheniformis* CGMCC 2876 contains plasmid pHY-P_3232_-*pgds* | This study |
| *Bl*/pP_2640_-*pgds* | *B. licheniformis* CGMCC 2876 contains plasmid pHY- P_2640_-*pgds* | This study |
| *Bl*/pP_2967_-*pgds* | *B. licheniformis* CGMCC 2876 contains plasmid pHY-P_2967_-*pgds* | This study |
| *Bl*/pP_2097_-*pgds* | *B. licheniformis* CGMCC 2876 contains plasmid pHY-P_2097_-*pgds* | This study |
| *Bl*/pP_3388_-*pgds* | *B. licheniformis* CGMCC 2876 contains plasmid pHY-P_3388_-*pgds* | This study |
| *Bl*/pP_3588_-*pgds* | *B. licheniformis* CGMCC 2876 contains plasmid pHY-P_3588_-*pgds* | This study |
| *Bl*/pP_3937_-*pgds* | *B. licheniformis* CGMCC 2876 contains plasmid pHY-P_3937_-*pgds* | This study |
| *Bl*/pP43-*pgds* | *B. licheniformis* CGMCC 2876 contains plasmid pHY-P43-*pgds* | This study |
| *Bl*/pP_spoVG_-*pgds* | *B. licheniformis* CGMCC 2876 contains plasmid pHY-P_spo_-*pgds* | This study |

**Table S3.** Transcriptome data of different promoters used in this study

| **Promoters** | **Transcriptome data in *B. licheniformis*** |
| --- | --- |
| P_2640_ | 36690.71 |
| P_3232_ | 12447.71 |
| P_2967_ | 10755.94 |
| P_3937_ | 10431.27 |
| P_3388_ | 6953.43 |
| P_2097_ | 6037.48 |
| P_3588_ | 5586.50 |
| P_3515_ | 5408.13 |

**Table S4**. The promoters used in this study

| Promoter | DNA sequence |
| --- | --- |
| P43 | TGATAGGTGGTATGTTTTCGCTTGAACTTTTAAATACAGCCATTGAACATACGGTTGATTTAATAACTGACAAACATCACCCTCTTGCTAAAGCGGCCAAGGACGCTGCCGCCGGGGCTGTTTGCGTTTTTACCGTGATTTCGTGTATCATTGGTTTACTTATTTTTTTGCCAAAGCTGTAATGGCTGAAAATTCTTACATTTATTTTACATTTTTAGAAATGGGCGTGAAAAAAAGCGCGCGATTATGTAAAAT |
| P_2640_ | GCGGTCATTAAAAATTTGTTTAAATAAGGTTATTTAAAATAATTATAATTTAGTATTGATTTTAATATTATATTTGTTATAATATAATTAACAAATGAAAACACGAGGAGGAAATATCA |
| P_3232_ | TAATATATTTCATCAACGATAATGAATTTTTCTTTATAATTATTATAAATAAATATTGTTTTTTTCTTGAGAAATGTTATCATTGTTTTGTAATTAAAATTTACGCGAGGTGATCCTTTG |
| P_2967_ | TTTCTGAATGCGATTTCAGTCGTTTTACATATTAATTGTAAGACAAAGAAGTATTGGAAAACAATTTCCACAAGATGTATATTTAATAATACAATAATTTTATTAAAAATTCATTGTAAACGAATGAAAATGGAGGAGTGAGGGCT |
| P_3937_ | TAAGCGAAGACGGCATCTGGAAAATCCGTGTTCACGGGTTAACGGCGTCATTTGAGGAGATCAGAACGGTTTTCAATGCAAAAATTAATAAAAAGGAGTTTTGATT |
| P_3388_ | TACTTAACAAAAAATTAACGGGCTTTCCCCTTGACAAAAAATATTTATTACTTGATAATTTGTATATAATTATAATAATTACAGAGAAATAAAGTTTACAATTTTTATTTCTCTTAAACTACATTCAGGAGGACGATATT |
| P_2097_ | TAAATGTTTGGAGAACAAGTTTTTATCATAACCCCTCCGCTATGCGCATAAATTTGTGGAGAAGCATATTTGTTATTCTCATCTGTTCGTTCACGTTTTTGCATAGGAGGGGAAAACG |
| P_3588_ | TGCCTCTTCATTAAAGTATAAATACTATTGTAAGATGAGAAAGTGAAACATTGCTTTCACTTTCTATCACTCTATATAACAAAAACTAACCTTTTTAAGGAGGATTTTCAGA |
| P_spoVG_ | AAATGAAAGCTTTATGACCTAATTGTGTAACTATATCCTATTTTTTCAAAAAATATTTTAAAAACGAGCAGGATTTCAGAAAAAATCGTGGAATTGATACACTAATGCTTTTATATAGGGAAAAGGTGGTGAACTACT |

**Table S5.** Primers used for PCR in this study

| **Primer name** | **Sequence of primer (5' to 3')^a^** |
| --- | --- |
| sfgfp-HindIII-F | AAAAACGCTTTGCCCAAGCTTATGAGCAAAGGAGAAGAACTTTTCA |
| sfgfp-kpnI-R | CTTCTCGAGACTAGTGGTACCTTATTTGTAGAGCTCATCCATGCC |
| sfgfp-kpnI-F | AGAGGAAAATCGGTACCATGAGCAAAGGAGAAGAACTTT |
| sfgfp-speI-R | TCTTCTCGAGACTAGTTTATTTGTAGAGCTCAT |
| rbs-GFP-F | GGGGGAGATTTGTATGAGCAAAGGAGAAGAACTTTTCA |
| GFP-R | CTTCTCGAGACTAGTGGTACCTTATTTGTAGAGCTCATCCATGCC |
| p3232-HindIII-F | AAAAACGCTTTGCCCAAGCTTTAATATATTTCATCAACGATAATGAATTTTT |
| p3232-rbs-R | GTTTCGGATCCCAAAGGATCACCTCGCGTAAA |
| p3588-HindIII-F | AAAAACGCTTTGCCCAAGCTTTGCCTCTTCATTAAAGTATAAATACTATTGT |
| p3588-rbs-R | CGGATCCTCTGAAAATCCTCCTTAAAAAGGTTA |
| p3388-HindIII-F | AAAAACGCTTTGCCCAAGCTTTACTTAACAAAAAATTAACGGGCTTT |
| p3388-rbs-R | TCGGATCCAATATCGTCCTCCTGAATGTAGTTTAAG |
| P 2640-F | AAAAACGCTTTGCCCAAGCTTGCGGTCATTAAAAATTTGTTTAAATAA |
| P 2640-R | CCCCTTTGTTGTTTCGGATCCTGATATTTCCTCCTCGTGTTTTCA |
| P 2967-F | AAAAACGCTTTGCCCAAGCTTTTTCTGAATGCGATTTCAGTCG |
| P 2967-R | CCCCTTTGTTGTTTCGGATCCAGCCCTCACTCCTCCATTTTCA |
| P 3937-F | AAAAACGCTTTGCCCAAGCTTTAAGCGAAGACGGCATCTGG |
| P 3937-R | CCCCTTTGTTGTTTCGGATCCAATCAAAACTCCTTTTTATTAATTTTTGC |
| P_2097_-F | AAAAACGCTTTGCCCAAGCTTTAAATGTTTGGAGAACAAGTTTTTATCA |
| P_2097_-R | TGTTTCGGATCCCGTTTTCCCCTCCTATGCAA |
| P_43_-F | AAAAACGCTTTGCCCAAGCTTTGATAGGTGGTATGTTTTCGCTTG |
| P_43_-R | CCCTTTGTTGTTTCGGATCCTTCATGTGTACATTCCTCTCTTACCTATA |
| P_spoVG_-F | AAAAACGCTTTGCCCAAGCTTAAATGAAAGCTTTATGACCTAATTGTG |
| P_spoVG_-R | TTCGGATCCAGTAGTTCACCACCTTTTCCCTATATAA |
| BamHI_2097_-RBS-F | GGAAAACGGGATCCGAAACAACAAAGGGG |
| 2640-RBS-F | GGAGGAAATATCAGGATCCGAAACAACAAAGGGG |
| 2967-RBS-F | GGCTGGATCCGAAACAACAAAGGGG |
| 3937-RBS-F | GGATCCGAAACAACAAAGGGG |
| RBS_3588_-F | GAGGATTTTCAGAGGATCCGAAACAACAAAGGGG |
| RBS3232-F | GATCCTTTGGGATCCGAAACAACAAAGGGG |
| RBS_3937_-F | GGATCCGAAACAACAAAGGGG |
| RBS_3388_-F | GAGGACGATATTGGATCCGAAACAACAAAGGGG |
| RBS_43_-F | ACACATGAAGGATCCGAAACAACAAAGGGGGAGA |
| RBS_spoVG_-F | GGTGAACTACTGGATCCGAAACAACAAAGGGG |
| RBS-sfgfp-R | TGCTCATACAAATCTCCCCCTTTGTTGTTT |
| RBS-pgds-R | GTTTGCCGCTTTTTTTATCAAACAAATCTCCCCCTTTGTTGTTTC |
| *pgdS*-rbs-F | GAAACAACAAAGGGGGAGATTTGTTTGATAAAAAAAGCGGC |
| *pgdS*-kpnI-R | CTTCTCGAGACTAGTGGTACCCTACTTAATTCTGACGCTTCCGG |
| ID-promoter-F | GAGCCTATGGAAAAACGCTTTGCCC |
| ID-promoter-R | TCCTATCACTCTTTTCTTCTCGAGA |
| *pgdS*-PamyL-F | AGAGGAAAATCGGTACCTTGATAAAAAAAGCGGCAAACAAAAAGT |
| *pgdS*-SpeI-R | TTCTTCTCGAGACTAGTCTACTTAATTCTGACGCTT |


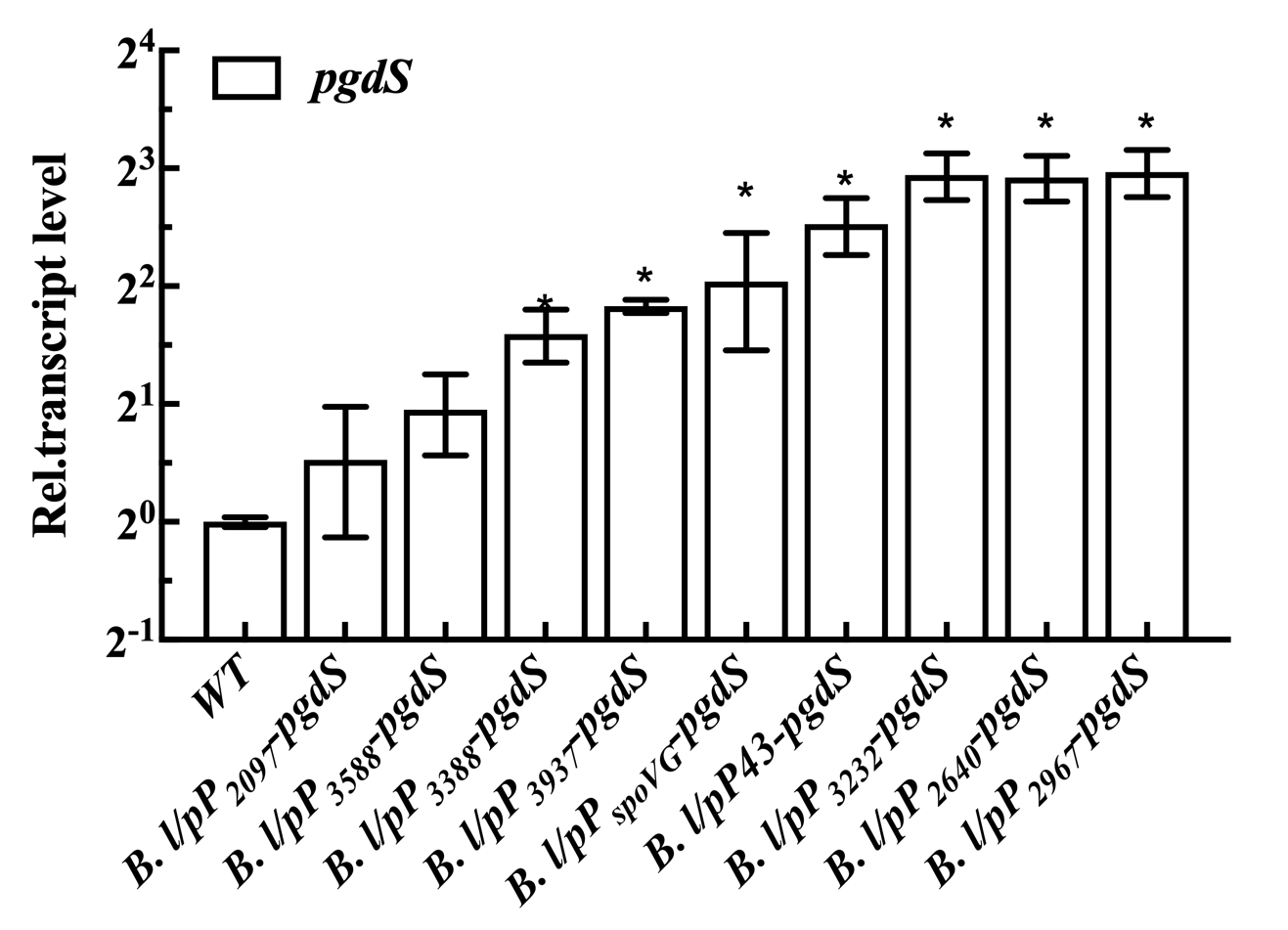


**FIG S1** The transcriptional levels of gene *pgdS* among strains with different promoters.

**FIG S2** A heatmap of the Pearson's correlation coefficients produced by comparing metabolites significantly affected by wild-type and *pgdS* engineered strains. Positive correlations are shown in red, negative correlations are shown in blue.

**
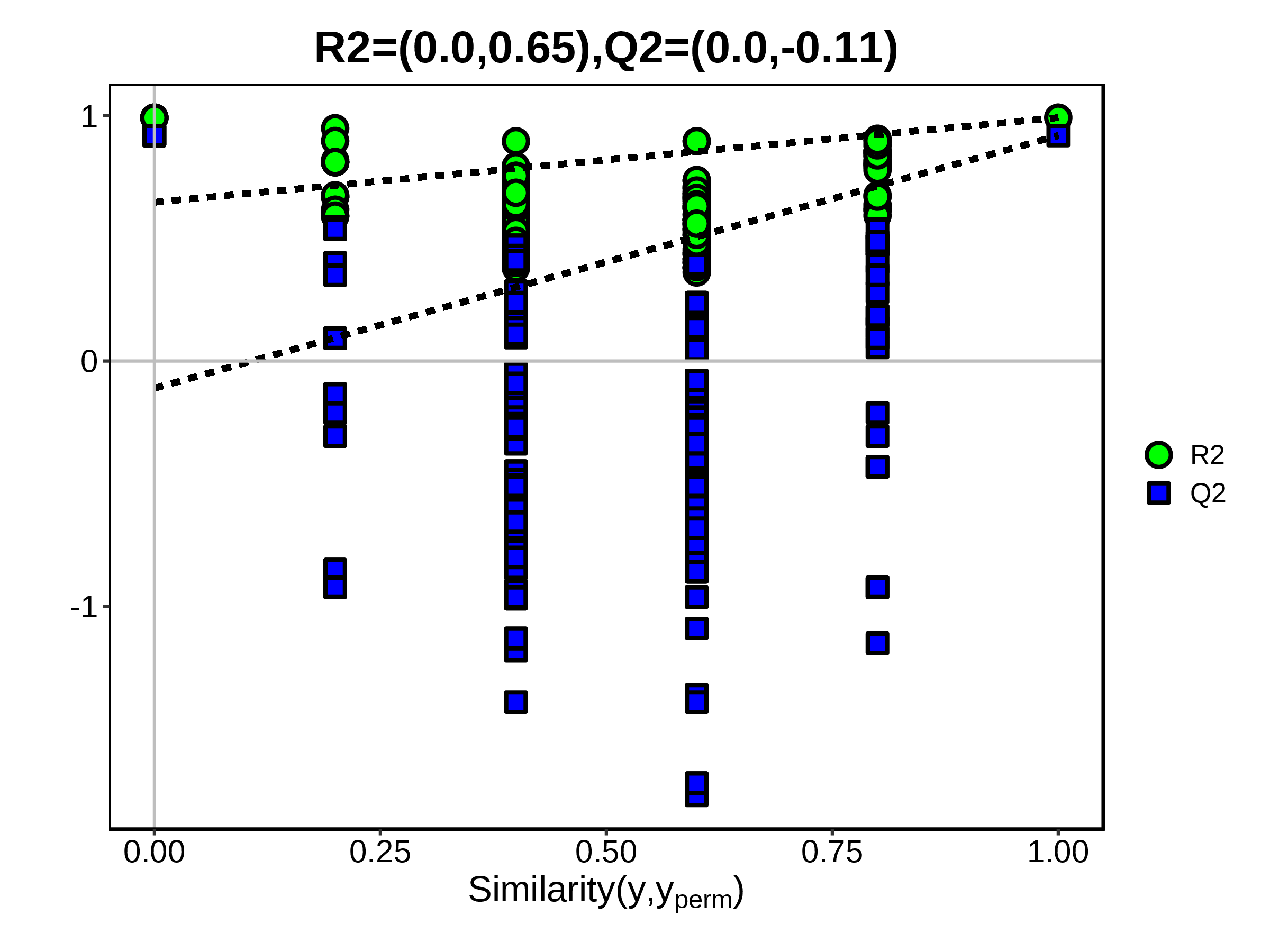
Fig. S3** Permutation test of cross validation (200 permutations) for the OPLS-DA model in wild-type and *Bl*/pP2967-pgdS strains.


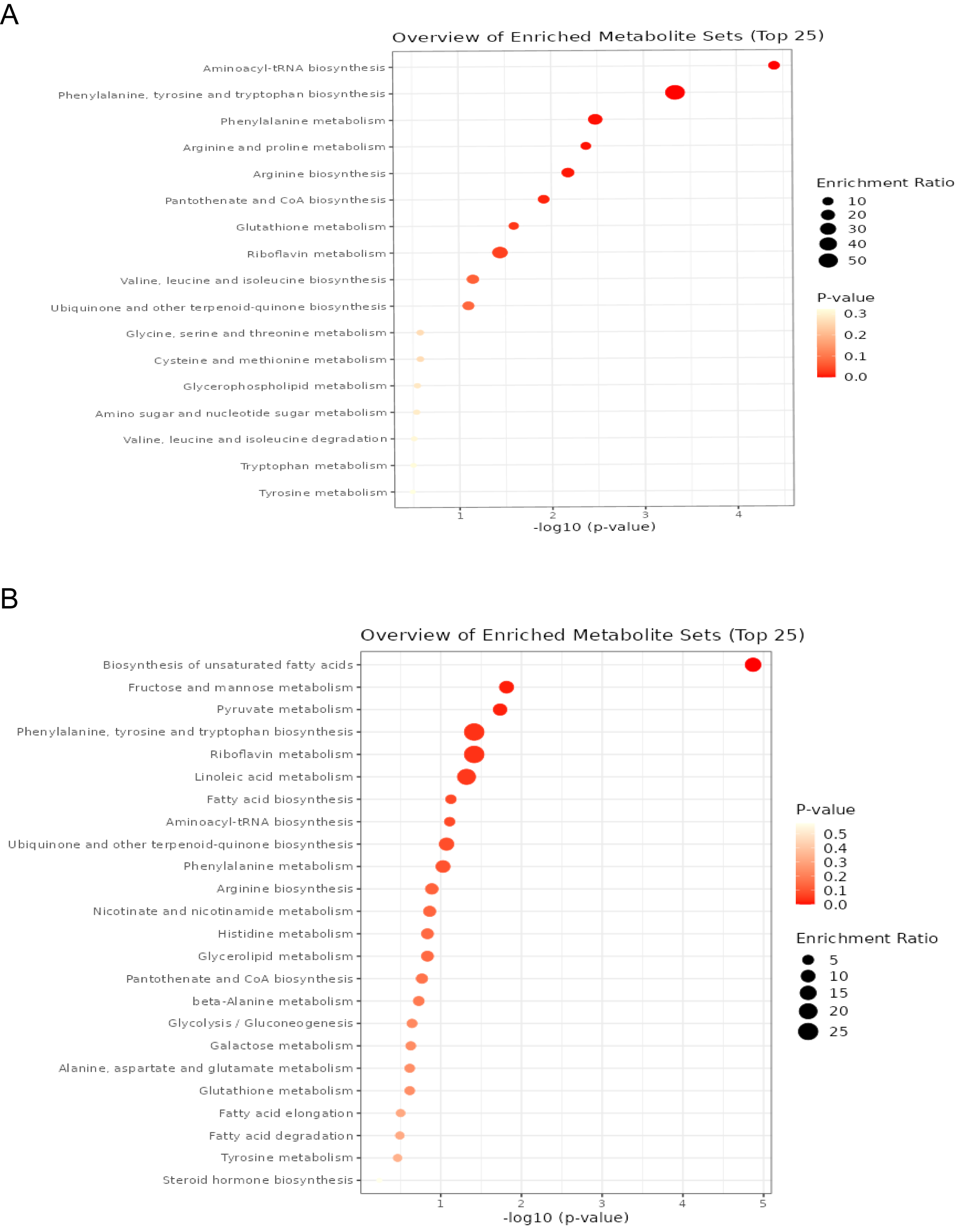


**FIG S4** The KEGG enrichment analysis of different metabolites screened in positive (A) and negatice (B) ion mode.

**
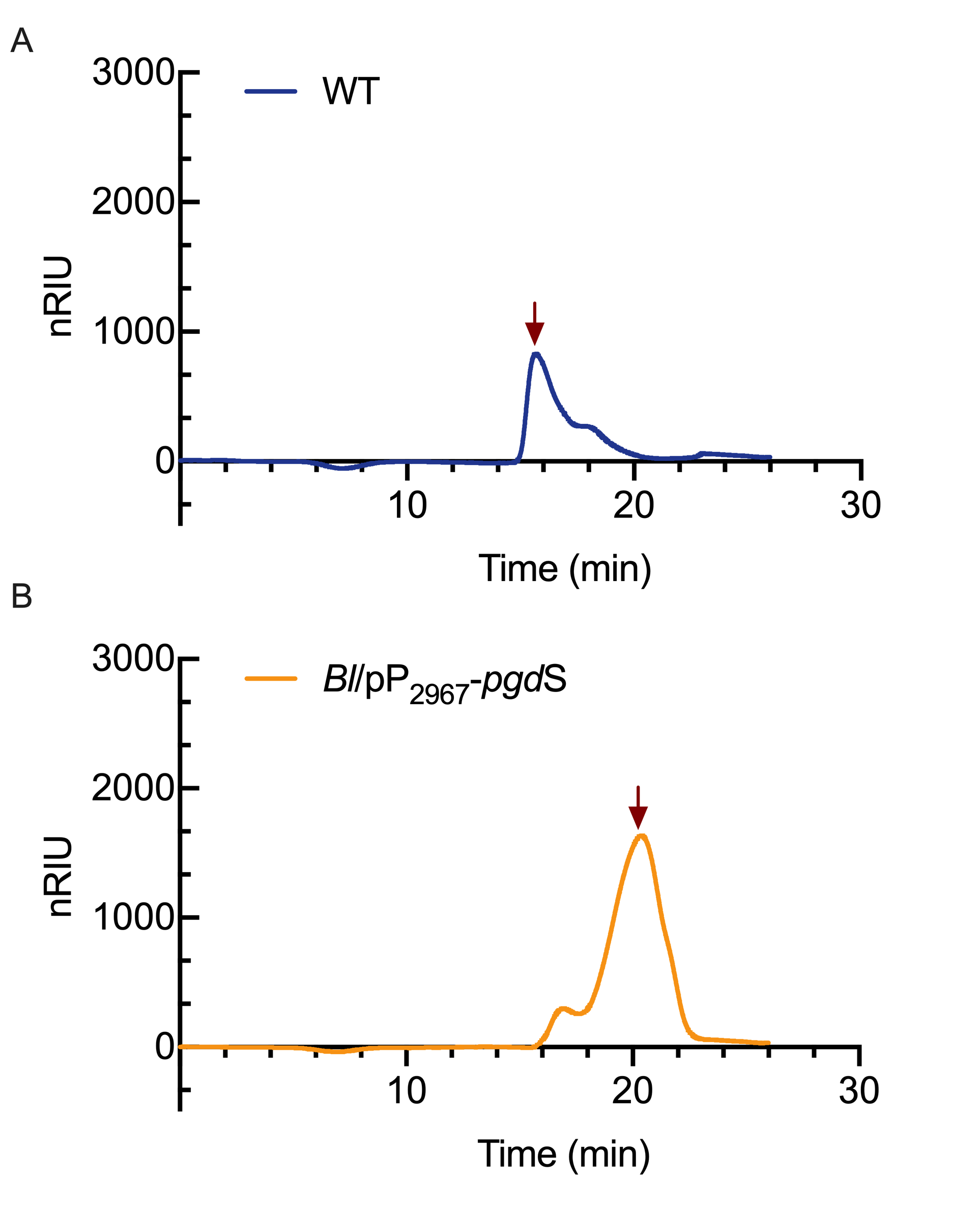
**

**FIG S5** Molecular weight analysis of γ-PGA between wild-type and *Bl*/pP2967-pgdS strains。


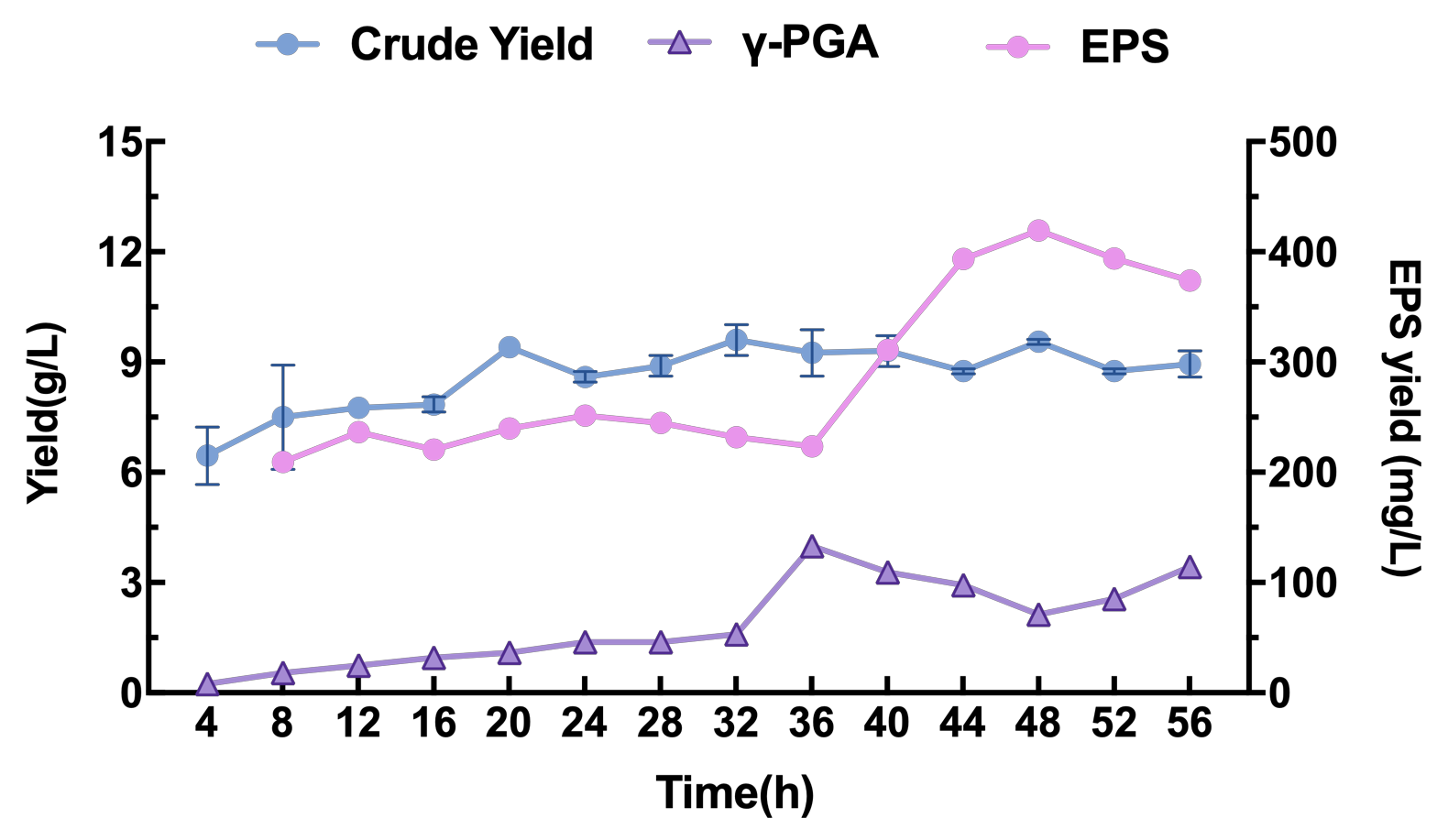


**FIG S6** The synthesis products of *B. licheniformis* CGMCC 2876 in 1.5-L fermenter.

Table S6 Identification of differential metabolites of wild-type and *Bl*/pP_2967_-*pgdS* strains.

| Metabolites | Compound ID | KEGG | log_2_FC | Pvalue | VIP |
| --- | --- | --- | --- | --- | --- |
| **Negative ion mode** |  |  |  |  |  |
| PFOA (Perfluorooctanoic Acid) |  |  | 6.163394427 | 0.016818519 | 1.533203798 |
| Salicylate | HMDB0000840 | C07588 | 3.294542297 | 0.006014906 | 1.568661859 |
| 4-Hydroxybenzoate | HMDB0000500 | C00156 | 3.294542297 | 0.006014906 | 1.568661859 |
| 3-Hydroxybenzoic acid | HMDB0002466 | C00587 | 3.294542297 | 0.006014906 | 1.568661859 |
| Linoleic acid | HMDB0000673 | C01595 | 1.935301904 | 0.015009115 | 1.467282385 |
| 10E,12Z-octadecadienoic acid | HMDB0005048 | C04056 | 1.935301904 | 0.015009115 | 1.467282385 |
| Estrone | HMDB0000145 | C00468 | 1.919035123 | 0.018725553 | 1.487336972 |
| PFEA (perfluoro-n-ethanoic acid) | HMDB0001056 | C00415 | 1.915637807 | 0.000205802 | 1.681289664 |
| Vaccenic acid | HMDB0003231 | C08367 | 1.890771723 | 0.014415489 | 1.464536082 |
| Oleate | HMDB0000207 | C00712 | 1.890771723 | 0.014415489 | 1.464536082 |
| Elaidic acid | HMDB0000573 | C01712 | 1.890771723 | 0.014415489 | 1.464536082 |
| (Z)-6-Octadecenoic acid | HMDB0002080 | C08363 | 1.890771723 | 0.014415489 | 1.464536082 |
| N-alpha-Acetyl-L-arginine | HMDB0004620 | na | 1.833349591 | 0.00473331 | 1.666375726 |
| FAD | HMDB0001248 | C00016 | 1.818899447 | 0.015030473 | 1.489545714 |
| Ibuprofen | HMDB0001925 | C01588 | 1.738881796 | 0.024498916 | 1.249190467 |
| 11-Aminoundecanoic acid | HMDB0032669 |  | 1.7344923 | 0.00848148 | 1.507723674 |
| Laurate | HMDB0000638 | C02679 | 1.673558469 | 0.007728097 | 1.518175081 |
| Dodecanoic acid | HMDB0000638 | C02679 | 1.673558469 | 0.007728097 | 1.518175081 |
| 10-Methylundecanoic acid |  |  | 1.673558469 | 0.007728097 | 1.518175081 |
| Gamma-Linolenic acid | HMDB0003073 | C00082 | 1.618494959 | 0.018951895 | 1.427665797 |
| Palmitate | HMDB0000220 | C00249 | 1.606973174 | 0.013331381 | 1.466733796 |
| Ricinoleic acid | HMDB0034297 | C08365 | 1.438838826 | 0.005485913 | 1.528733721 |
| 2,5,7,8-Tetramethyl-(2-carboxyethyl)-6-hydroxychroman |  |  | 1.388094744 | 0.014282028 | 1.449959419 |
| Stearate | HMDB0000827 | C01530 | 1.244309449 | 0.025741122 | 1.333812136 |
| Ethyl hexadecanoate | HMDB0029811 |  | 1.244309449 | 0.025741122 | 1.333812136 |
| Aspartic acid | HMDB0000191 | C00049 | 1.199551794 | 0.005936399 | 1.527937638 |
| Dodecylbenzenesulfonic acid | HMDB0034266 | C08271 | 1.199210822 | 0.003094403 | 1.586834463 |
| Naringin | HMDB0002927 | C09789 | -1.311513063 | 0.044601942 | 1.181572311 |
| Lapachol |  |  | -1.317751772 | 0.004052006 | 1.297212422 |
| Equol | HMDB0002209 | C14131 | -1.317751772 | 0.004052006 | 1.297212422 |
| N-acetylleucine | HMDB0011756 | C02710 | -1.433974986 | 0.000552419 | 1.405337469 |
| Maleamate | METPA0190 | C01596 | -1.626882306 | 0.013695613 | 1.189383063 |
| Tyrosine | HMDB0000158 | C00082 | -1.660417385 | 0.00238155 | 1.354030477 |
| xylulose |  |  | -1.824151803 | 0.001343202 | 1.356149043 |
| Linderane |  |  | -1.844848444 |  | 1.646889151 |
| D-Lactic acid | HMDB0001311 | C00256 | -2.005987214 | 0.00469587 | 1.46851369 |
| Lactate | HMDB0000190 | C00186 | -2.005987214 | 0.00469587 | 1.46851369 |
| Glyceraldehyde | HMDB0001051 | C02154 | -2.005987214 | 0.00469587 | 1.46851369 |
| 2-Isopropylmalic acid | HMDB0000402 | C02504 | -2.010552479 | 0.025220735 | 1.278334112 |
| D-(-)-Erythrose |  |  | -2.116597465 | 0.000887304 | 1.38005196 |
| Cys-Gly | HMDB0000078 | C01419 | -2.318058562 | 0.042289064 | 1.131103442 |
| Sorbitol | HMDB0000247 | C00794 | -2.401954358 | 0.049336281 | 1.336667331 |
| Mannitol | HMDB0000765 | C00392 | -2.401954358 | 0.049336281 | 1.336667331 |
| D-Sorbitol | HMDB0000247 | C00794 | -2.401954358 | 0.049336281 | 1.336667331 |
| Homoserine | HMDB0000719 | C00263 | -2.452439018 | 0.003712314 | 1.315739271 |
| N-Acetyl-L-lysine | HMDB0000446 | C12989 | -2.806632889 | 0.005251338 | 1.530064137 |
| N-alpha-acetyllysine | HMDB0000446 | C12989 | -2.806632889 | 0.005251338 | 1.530064137 |
| **Positive ion mode** |  |  |  |  |  |
| Moperone |  |  | 2.960525435 | 0.000108218 | 1.545140967 |
| Pantothenate | HMDB0000210 | C00864 | 2.827813061 | 0.001014327 | 1.445877326 |
| 4-Methylpyrazole | HMDB0015344 | C07837 | 2.130053963 | 0.00000211 | 1.548416105 |
| Tyrosine | HMDB0000158 | C00082 | 2.068162863 | 0.003458271 | 1.278773948 |
| p-aminocinnamic acid |  |  | 2.068162863 | 0.003458271 | 1.278773948 |
| Anthranilate | HMDB0001123 | C00108 | 2.068162863 | 0.003458271 | 1.278773948 |
| 4-Aminobenzoate | HMDB0001392 | C00568 | 2.068162863 | 0.003458271 | 1.278773948 |
| 3-Pyridylacetic acid | HMDB0001538 |  | 2.068162863 | 0.003458271 | 1.278773948 |
| Trigonelline | HMDB0000875 | C01004 | 1.974617566 | 0.00931936 | 1.190415693 |
| FAD | HMDB0001248 | C00016 | 1.683632753 | 0.000937664 | 1.516627876 |
| 2,6-Dimethylnaphthalene | HMDB0030769 | NA | 1.575473611 | 0.000661837 | 1.382225378 |
| Putrescine | HMDB0001414 | C00134 | 1.57411539 | 0.019078782 | 1.241140623 |
| N-Acetyl-L-lysine | HMDB0000206 | C02727 | 1.539371296 | 0.0000631 | 1.579064904 |
| Chlorotoluron |  |  | 1.518851385 | 0.007012922 | 1.238796555 |
| Methotrexate | HMDB0014703 | C01937 | 1.4517043 | 0.010736124 | 1.144369019 |
| L-Proline | HMDB0000162 | C00148 | 1.4517043 | 0.010736124 | 1.144369019 |
| Arginine | HMDB0000517 | C00062 | 1.37998226 | 0.003173241 | 1.321913886 |
| 5-Methylthioadenosine | HMDB0001173 | C00170 | 1.306016403 | 0.002594429 | 1.394069765 |
| Glycerol-Myristate |  |  | 1.272989644 | 0.0000173 | 1.33446298 |
| N-Acetyl-D-mannosamine | HMDB0013112 | C01170 | 1.266905076 | 0.005865943 | 1.370589393 |
| Citrulline | HMDB0000904 | C00327 | 1.242324325 | 0.003119461 | 1.228513576 |
| N-α-Acetyl-L-arginine | HMDB0004620 | NA | 1.242324325 | 0.003119461 | 1.228513576 |
| L-Methionine sulfoxide | HMDB0002005 | C02989 | 1.189631853 | 0.0000225 | 1.350559096 |
| Choline | HMDB0000097 | C00114 | 1.182671827 | 0.000151819 | 1.554175272 |
| PEG-10mer Ammonium adduct |  |  | 1.027157542 | 0.037348355 | 1.007047227 |
| Phenylalanine | HMDB0000159 | C00079 | -1.021133043 | 0.013148781 | 1.350048021 |
| Bornaprine |  |  | -1.127089853 | 0.009854848 | 1.542868325 |
| Doxycycline Tautomer |  |  | -1.23814936 | 0.001839868 | 1.388465918 |
| Chlordimeform |  |  | -1.261131443 | 0.009722235 | 1.22742564 |
| 1-Naphthylacetic Acid | HMDB0032708 | C13014 | -1.335136768 | 0.000521721 | 1.501424916 |
| Valine | HMDB0000883 | C00183 | -1.958500077 | 0.013464875 | 1.123168792 |
| Oxoproline | HMDB0000267 | C01879 | -2.543959893 | 0.001311455 | 1.572340491 |
| Neburon |  |  | -2.543959893 | 0.001311455 | 1.572340491 |
| Tetracycline | HMDB0014897 | C06570 | -4.061801494 | 5.89E-08 | 1.54195247 |
